# Multimodal sensor-based response mechanisms of drivers under adverse weather conditions using EEG spectral entropy and prediction entropy

**DOI:** 10.3389/fnins.2026.1919198

**Published:** 2026-09-09

**Authors:** Yi Tian, Jianping Hu, Hao Ding, Binhe Yang, Jialin Hu, Yuting Liu, Yu Ding

**Affiliations:** 1 School of Traffic and Transportation Engineering, Xinjiang University, Urumqi, China; 2 Xinjiang Key Laboratory of Green Construction and Smart Traffic Control of Transportation Infrastructure, Xinjiang University, Urumqi, China

**Keywords:** adverse weather, cognitive workload, complex system, driver state, information complementarity, multimodal sensors, neuroergonomics, prediction entropy

## Abstract

**Introduction:**

Whether rain, snow, and fog elicit qualitatively different driver-state dynamics remains unclear.

**Methods:**

Thirty licensed drivers completed a simulator experiment under clear, rain, snow, and fog conditions, during which subjective scales, electrocardiograms, electroencephalograms, and vehicle data were acquired simultaneously. Six response pathways were constructed to characterize subjective load, operational fluctuation, conservative control, physiological arousal, electroencephalographic response, and system uncertainty. C4 spectral entropy gauge neural complexity, class-wise optimized reliability fusion prediction entropy measured system uncertainty in multimodal sensor responses, and within-subject centering, together with distance correlation, revealed how information is structured across modalities.

**Results:**

Among out-of-fold predictions over 6,456 windows, the class-wise optimized reliability fusion model achieved 91.9% accuracy and Macro-F1 of 0.919. All three adverse weather types significantly increased the subjective load, yet the dominant pathways differed. Rainfall increased cognitive load, snow led to synchronized increases in neural complexity and system uncertainty, and fog induced conservative compensation under visual restriction. Snow produced the strongest system-level response [class-wise optimized reliability fusion prediction entropy: *r* = 0.79, 95% confidence interval (CI): (0.62, 0.87); C4 spectral entropy: *r* = 0.53, 95% CI (0.21, 0.79)]. The four modalities formed an information-complementary structure.

**Discussion:**

This study elucidates the information dynamics of driving states under adverse weather conditions and demonstrates that rain, snow, and fog occupy distinct regions within the information space, thereby providing a sensor-informed foundation for driver-state monitoring and graded warning systems in intelligent vehicles.

## 1 Introduction

Rainfall, snow, and fog substantially challenge human cognitive-motor performance and the neural mechanisms underlying driver-state monitoring. Qi and Sun (2023) reported that adverse weather conditions contribute to approximately 20% of traffic accidents. Hjelkrem and Ryeng (2016) found that snow-covered and contaminated surfaces reduce road adhesion and increase crash rates to 13 times the non-snow baseline. Chang et al. (2019) confirmed that fog-induced visibility loss is a leading cause of severe freeway crashes. Subjective load, cardiac activity, electroencephalogram (EEG) dynamics, and vehicle control constitute a strongly coupled system, and no single indicator can linearly depict the internal state. However, whether rain, snow, or fog triggers qualitatively different state changes remains unclear because information-theoretic evidence remains limited.

Previous studies have explored this issue from three perspectives: driving behavior, neurophysiology, and fusion methods. At the driving-behavior level, Chang et al. (2019) observed that fog prompts drivers to reduce speed; however, increased speed dispersion persists, indicating that behavioral adjustment alone cannot fully offset visibility loss. Wang et al. (2020) used heart-rate variability to demonstrate that low fog-induced visibility levels increase the mental load of drivers as speed increases. Lin and Chen (2024) further noted that low visibility substantially weakens takeover capability in emergency car-following. These studies predominantly focused on single-weather or single-modality descriptions and did not compare the three weather types as an integrated system of perception, cognition, and behavior control.

At the neurophysiological level, Kamti et al. (2023) used EEGs to show that attention, fatigue, and mental load significantly fluctuate under rain and fog, with large individual differences. Ni et al. (2024) reviewed the effects of environmental stress in altering driver state, noting that multi-source environmental loads systematically change physiological response patterns; however, they did not analyze the distinctions among rain, snow, and fog. Sikander and Anwar (2018) surveyed multimodal fatigue detection and concluded that a single sensor cannot cover all facets of the driving state. Conventional linear metrics, such as the power spectrum, assume signal stationarity, whereas EEGs and electrocardiograms (ECGs) exhibit significant deviation from stationarity under adverse weather conditions. Perrey (2025) argued that conventional linear EEG analysis fails to capture the inherent complex dynamics of neural signals, whereas entropy, which is a model-free measure, provides a more effective description of such nonlinear fluctuations. Yang et al. (2025) fused multiple entropy features to predict driving fatigue, confirming that entropy metrics are useful for driving-state assessment. Hadra et al. (2022) further showed that EEG temporal complexity encodes alertness changes. Guo et al. (2015) introduced EEG spectral entropy to driving mental-load recognition early on, extracting θ, α, and β power via fast Fourier transform (FFT) and computing Shannon entropy to construct a load-recognition model; Guo et al. (2016) subsequently combined spectral entropy with support vector machines and verified the sensitivity of entropy to mental load. However, these studies mostly targeted a single state or single modality and did not extend entropy analysis to a multi-weather, multimodal comparison.

In recent years, multimodal fusion has become the primary method for robust driving state monitoring. Wang et al. (2023) fused EEG, ECG, and vehicle-image data via a feedback mechanism for real-time fatigue recognition, achieving an accuracy exceeding 90% and confirming that multi-source physiological sensors are complementary. Wu et al. (2023) compared EEGs, ECGs, and electrooculograms (EOGs) in drowsiness detection, finding that different modalities are sensitive at different time windows, which indicates information complementarity. Soumya and Mythili (2026) fused multimodal physiological signals via graph neural networks for cognitive distraction detection, further confirming the necessity of multi-source fusion. Zhang et al. (2025) proposed transformer-based multimodal framework with uncertainty quantification (MU-Net), a framework that fuses EEGs and EOGs via an uncertainty-weighted gating mechanism to dynamically integrate cross-modal information and output predictive uncertainty; they treated uncertainty as a signature of modality reliability rather than a model flaw, offering an interpretable fusion example for driving-state monitoring. However, these studies mostly rely on ideal weather conditions, and a few have systematically validated heavy rain, heavy snow, or dense fog conditions. Moreover, these studies focus on whether multimodal features can recognize states, while leaving open how system uncertainty changes across weather types and how modalities interact at the mechanism level.

Two major gaps emerged in prior studies. First, most studies have focused on a single weather type or modality and do not systematically compare driver states under rain, snow, and fog conditions as a whole. If rain, snow, and fog act on the driver through different dominant pathways, a monitoring system cannot rely on one global threshold but must be calibrated for each weather type. Second, existing studies largely address whether multimodal features are recognizable; however, they lack an information-theoretic characterization of how modalities interact across weather types and how system uncertainty changes. This information structure, not the recognition score itself, is the structure on which sensor selection and fusion-weight design depend on in safety-critical monitoring.

This study makes a novel contribution by employing EEG spectral entropy and class-wise optimized reliability fusion (CORF) prediction entropy as information-theoretic tools to characterize driver-state dynamics under adverse weather conditions. Unlike conventional behavioral or physiological analyses that assume linearity and stationarity, entropy captures the inherent nonlinear fluctuations of physiological signals under adverse weather and reveals how system uncertainty changes across weather types. Previous EEG studies used spectral entropy mainly as a classification feature under normal driving (Guo et al., 2015, 2016); instead, we used central electrode position (C4) spectral entropy as a descriptive measure of neural complexity and paired it with CORF prediction entropy as a state-boundary measure in a multi-weather, multimodal within-subject comparison.

Therefore, this study targets the limitation that the driver state under adverse weather conditions cannot be captured by a single metric. The analysis employs EEG spectral entropy and model prediction entropy to measure information complexity and system uncertainty; analyzes how rain, snow, and fog alter subjective feelings, cardiac responses, EEG dynamics, and driving behavior through different pathways; and reveals the extent to which these four information sources exhibit redundancy or complementarity. We recruited 30 licensed drivers for a simulator experiment across clear, rain, snow, and fog conditions; acquired multimodal sensor streams including subjective scales, ECGs, EEGs, and vehicle data simultaneously; and used the previously developed CORF model to generate window-level prediction entropy and confidence. These data were aggregated after cross-validation and entered into weather-level statistical comparisons, aiming to provide an information-theoretic mechanism basis for driver-state monitoring under adverse weather conditions. We test herein three hypotheses: (a) H1: all three adverse weather types increase subjective load but through different dominant pathways (rain: cognitive-autonomic; snow: neural-complexity and system-uncertainty; fog: conservative control); (b) H2: C4 spectral entropy and CORF prediction entropy jointly separate the three weather types in the information space, and (c) H3: the four modalities form an information-complementary structure with nonlinear cross-modal associations.

## 2 Materials and methods

### 2.1 Experimental design and data acquisition

**FIGURE 1:**
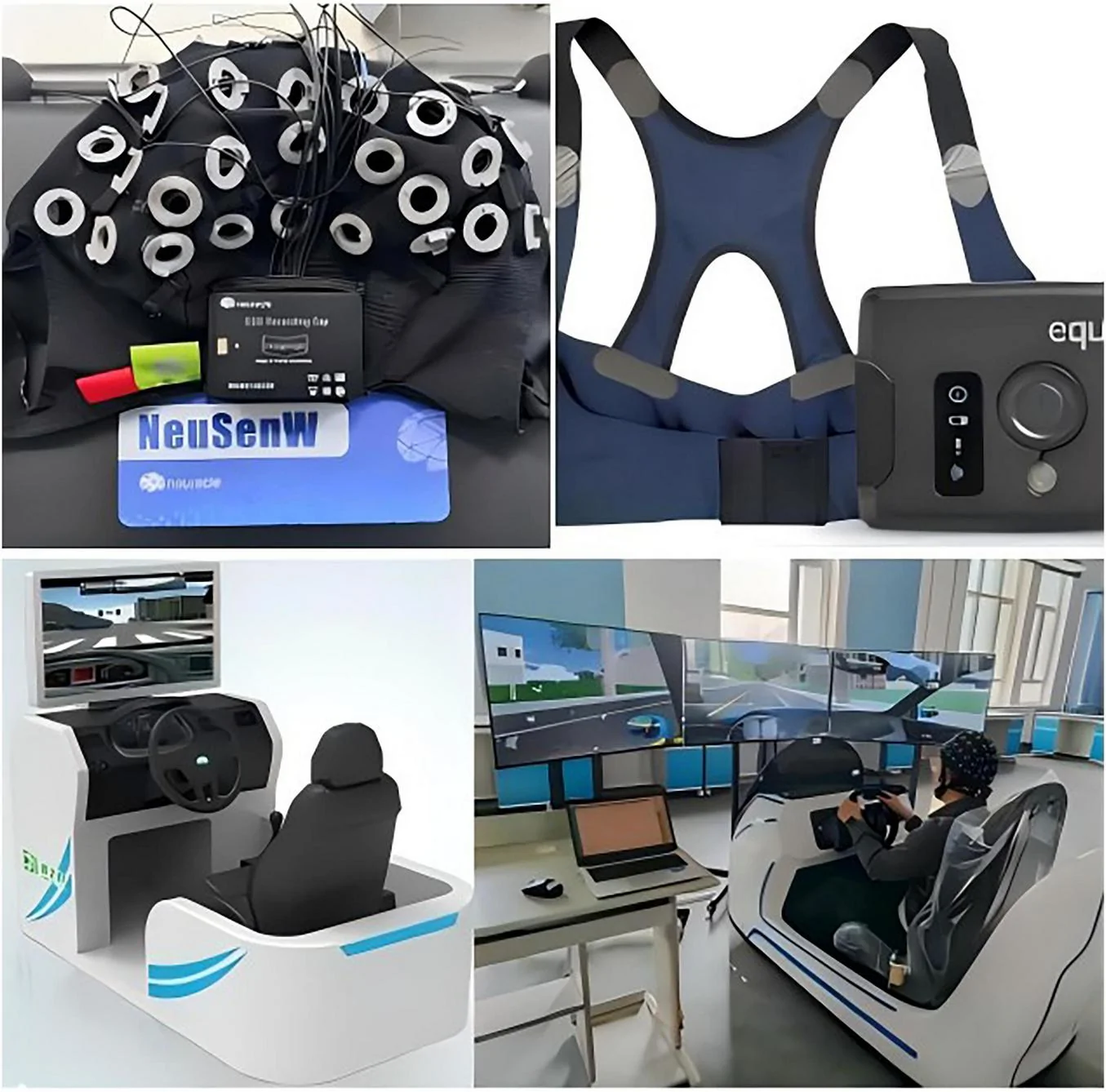
Experimental setup. Left: NeuSen W 8-channel EEG system; middle: Equivital physiological monitoring vest; right: driving simulator with a Logitech G29 steering wheel.

An experiment was conducted using a Maike driving simulator with a Logitech G29 steering wheel (Logitech International S.A., Lausanne, Switzerland) (Figure 1). The scenario involved a typical urban road with straight and curved sections, 3.5-m lane width, and 60 km/h speed limit, with a fixed level of surrounding traffic, including other vehicles, bicycles, and pedestrians, held identical across weather conditions so that weather was the only manipulated factor; the driving task was uniform (speed-limit driving with lane keeping). We set four weather conditions: clear, rain, snow, and fog. Clear served as the control, whereas rain, snow, and fog were adverse weather conditions. Visibility parameters followed the China Meteorological Administration scale: heavy rain and dense fog at 200–500 m, and heavy snow below 500 m. To control for order effects, we arranged the four weather tasks in a Latin square design in a randomized order. After equipment fitting and a signal check, participants completed a 5–10 min familiarization drive in clear weather conditions, followed by a 2-min resting baseline recording during which the eyes were closed. Each weather task lasted 6 min. After each task, participants were exposed to clear weather conditions for a 3-min recovery drive. Then, they parked for a 2-min break to complete the National Aeronautics and Space Administration (NASA) task load index (NASA-TLX) and Karolinska Sleepiness Scale (KSS). The full session lasted 50–60 min per participant.

Thirty licensed drivers participated in the study (20 males, 10 females; aged 20–45 years; driving experience < 1–5 years; driving frequency from occasional to regular). Inclusion criteria were a valid C1 (or above) license; normal or corrected-to-normal vision without color blindness; and no history of neurological disease, brain injury, or simulator sickness. Participants abstained from alcohol, coffee, and strong tea for 24 h beforehand and had sufficient sleep. Each participant drove through all four weather tasks in sequence and completed the NASA-TLX and KSS after each task.

We simultaneously collected three data streams. EEGs were recorded at eight positions (F4, C3, Fz, Cz, F3, Oz, C4, and Pz) using a NeuSen W wireless system (Neuracle Technology Co., Ltd., Changzhou, China; 256 Hz; Ref/Gnd electrodes). Electrocardiography and respiration were captured using an Equivital system (Hidalgo Ltd., Cambridge, United Kingdom) at 256 Hz (Lead1/Lead2), which yielded the heart rate, RR intervals, and breathing rate. Vehicular data, including speed, acceleration, steering angle, throttle opening, brake opening, and lateral deviation, were obtained directly from the simulator. All streams were recorded on a shared synchronization platform with unified timestamps; task events and weather switches were logged with a synchronized video record. Windowing used 10-s windows (5-s step) for all streams, and ECG-derived indices were computed on the same windows as the EEG. Ethical review and approval were waived for this study owing to the use of a non-invasive driving simulator with no physical or psychological risks beyond normal daily activities. All participants provided written informed consent before the experiment.

**FIGURE 2:**
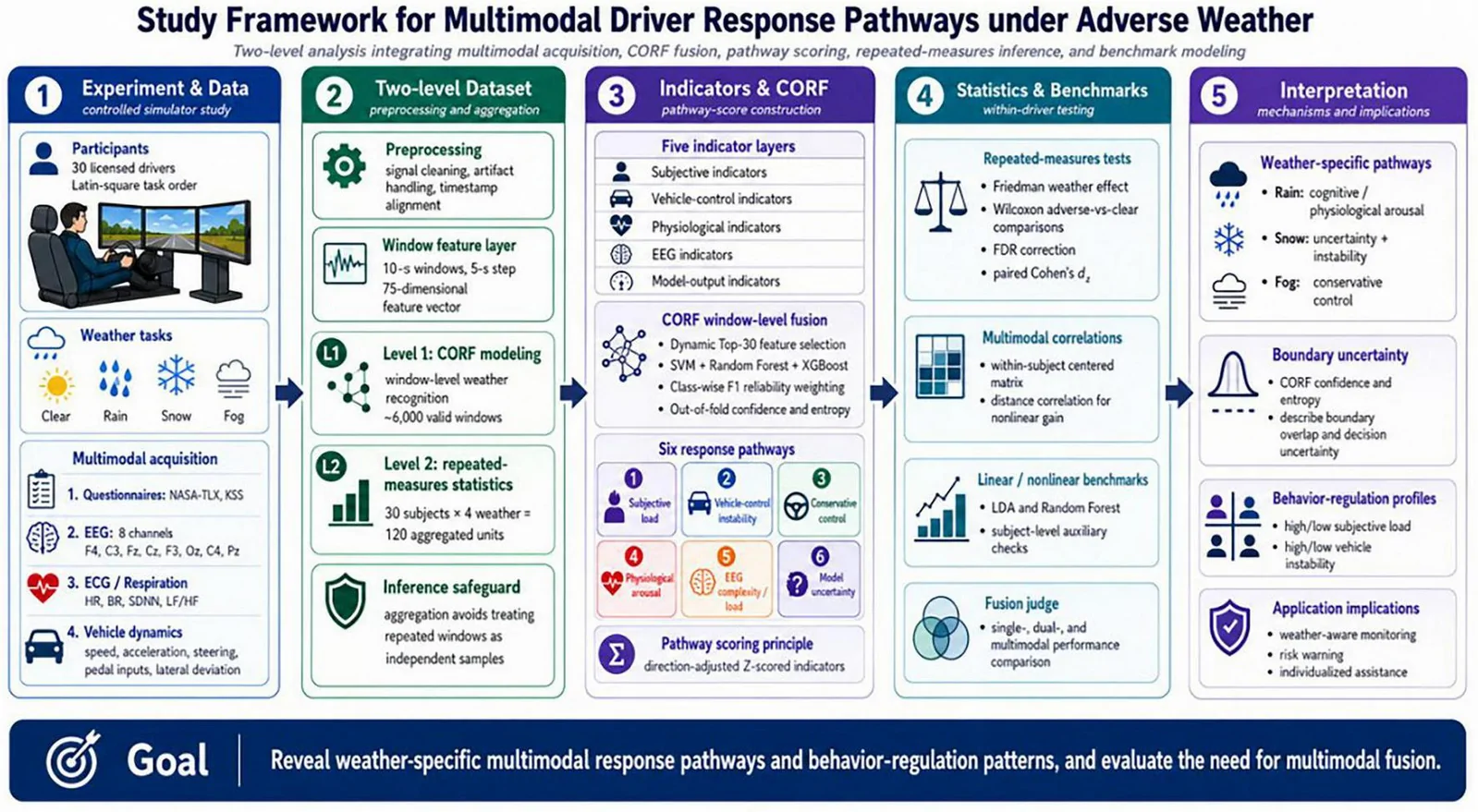
Study framework for multimodal driver-response pathway analysis under adverse weather.


We analyzed the data on two timescales (Figure 2). First, we segmented the data into 10-s windows and extracted 75-dimensional feature vectors for the fusion model, yielding approximately 6,000 valid windows. Second, we averaged the windows of each participant under each weather condition to obtain 120 aggregated data points (30 participants × 4 weather conditions) for a weather-level statistical comparison. Before aggregation, we first computed the per-indicator means within each participant × weather cell [as expressed by Equation 1], and then applied the following Z-score standardization: participant × weather cell [as expressed by Equation 2]:

$${\bar{\text{x}}}_{\text{ij}}^{\left(k\right)}=\frac{1}{{\text{n}}_{\text{ij}}}\sum_{\text{q}=1}^{{\text{n}}_{\text{ij}}}{\text{x}}_{\text{ijq}}^{\left(k\right)},\text{i}=1,,30;\text{j}\in \left\{\mathrm{Clear},\mathrm{Rain},\mathrm{Snow},\mathrm{Fog}\right\},$$ (1)

$${\text{z}}_{\text{ij}}^{\left(k\right)}=\frac{{\bar{\text{x}}}_{\text{ij}}^{\left(k\right)}-\mu_{\text{k}}}{\sigma_{\text{k}}},$$ (2)

where ${\bar{\text{x}}}_{\text{ij}}^{\left(\text{k}\right)}$ denotes the mean of indicator k for participant i under weather j, and μ_k_ and σ_k_ are the overall mean (M) and standard deviation (SD), respectively, of that indicator across all 120 aggregated points.

### 2.2 Indicator definition and data preprocessing

Subjective load indicators include the NASA-TLX total score and KSS rating. The NASA-TLX contains four subdimensions—mental demand, temporal demand, effort, and frustration—each scored from 0 to 20, with a total outcome in the range of 0–80. The KSS is a 1–9 fatigue scale (The other two standard subdimensions, physical demand and own performance, were omitted because the simulator task involved minimal physical effort and no external performance feedback).

Cardiac response indicators include heart rate mean, breathing rate, standard deviation of normal-to-normal intervals (SDNN), and low frequency/high frequency (LF/HF) ratio, reflecting autonomic regulation and cardiovascular arousal. Among common heart rate variability (HRV) indices, only SDNN and LF/HF were retained. With the use of 10-s windows, separate LF and HF power estimates are unstable, whereas SDNN and the segment-level LF/HF ratio remain interpretable when ultra-short-term norms are used (Shaffer et al., 2020); root mean square (RMS) SD was excluded as highly collinear with SDNN. Longer-window HRV analysis constitute future work.

EEG-activity indicators include the F4-channel (θ+α)/β ratio and C4-channel spectral entropy. EEG signals were preprocessed with the EEGLAB toolbox (Swartz Center for Computational Neuroscience, University of California, San Diego, La Jolla, CA, United States): 1–40 Hz band-pass filtering, baseline correction, and independent component analysis to remove ocular and muscular artifacts. The C4 spectral entropy captures EEG complexity: we applied FFT to the C4 signal; extracted power spectral density at δ, θ, α, β, and γ bands (γ: 30–40 Hz, bounded by the 40-Hz low-pass filter); normalized each band’s power into a probability distribution; and then computed Shannon entropy using Equation 3:


SE=-∑p⁢(f)⁢log⁡p⁢(f). (3)

higher spectral entropy indicates a more dispersed power distribution and more complex EEG activity, whereas a lower entropy indicates more monotonic activity. This procedure followed that described by Guo et al. (2015).

ECG signals were preprocessed with a 1–40 Hz bandpass filter; R-peaks were detected using the Pan–Tompkins algorithm (Pan and Tompkins, 1985), and RR intervals were visually inspected for ectopic beats. The vehicle control indicators included speed mean, speed standard deviation, RMS acceleration, steering standard deviation, and absolute lateral deviation. We further constructed an operational instability index by averaging the four fluctuation indicators—speed standard deviation, RMS acceleration, steering SD, and absolute lateral deviation—after standardization; higher scores indicated larger control fluctuations.

The model output indicators were derived from the CORF fusion results, covering confidence, prediction entropy, and prediction accuracy. The prediction entropy is the Shannon entropy of the fused probability distribution. We define it as an index of state-boundary ambiguity: the degree to which the multimodal response pattern of a window falls in the overlapping region between weather categories, under a fixed, validated classifier. It thus reflects a property of the driver–vehicle system rather than the classifier’s own reliability (Huseljic et al., 2020); a higher value indicates less separable driving states under that weather. Section 3.2 verifies this pattern among correctly classified windows; the Limitations section addresses its model dependence. Prediction entropy is therefore an information-theoretic metric of driving-state stability, not a by-product of the model output.

We constructed six composite indicators along the aforementioned dimensions: subjective load, operational fluctuation, conservative control, cardiac response, EEG response, and prediction entropy. Each composite indicator was obtained by averaging its constituent raw indicators after standardization, with direction coefficients applied; higher scores indicated a stronger response along that dimension. These composite indicators are formative pathway scores; internal-consistency coefficients do not apply, as constituent indicators may capture distinct pathway facets (e.g., the two EEG indicators whose within-modality correlation is close to zero after centering).

### 2.3 Fusion model and uncertainty measurements

To identify weather states from the multimodal window features, we used a previously developed CORF model as a multimodal analysis tool. Inspired by the Reward-Penalty Weighted Ensemble proposed by Nandi et al. (2022), CORF replaces global weights with class-wise reliability weights such that difficult weather categories receive a fuller fusion expression. CORF takes 75-dimensional features from 10-s windows as input and outputs four-class weather probabilities. We adopted within-subject, stratified five-fold cross-validation: in each fold, we dynamically selected the top-30 features via random-forest importance from the training set, trained three base classifiers—support vector machine (SVM), random forest (RF), and extreme gradient boosting (XGBoost)—and then computed the reliability weights from the validation set’s F1-scores of each class to complete probability fusion. Window-level prediction entropy, confidence, and accuracy were obtained from out-of-fold predictions during cross-validation and were then aggregated by participant weather into 120 summary data points, avoiding circular overlap between training and explanatory data and entering subsequent weather-level statistical comparisons.

Let M = {*SVM*, *RF*, *XGBoost*} be the base classifier set, and the probability that model m is assigned to class c for window x is p_m_(c|x). Let F_m,c_ be the F1 score of model m in class c in the validation set; the model-class reliability weight is expressed by Equation 4:


$$\rho_{m,c}=\frac{F_{m,c}+\varepsilon }{\sum_{l\in M}\left(F_{l,c}+\varepsilon \right)},\varepsilon =10^{-6}.$$ (4)

Fusion probability first computes unnormalized scores and normalizes them as expressed by Equation 5 and Equation 6:


$${\text{s}}_{\text{c}}\,\left(x\right)=\sum_{\text{m}\in \text{M}}\rho_{m,c}\cdot {\text{p}}_{\text{m}}\,\left(c|x\right),$$ (5)

$$P\,\left(c|x\right)=\frac{{\text{s}}_{\text{c}}\,\left(x\right)}{\sum_{\text{k}\,1}^{\text{K}}{\text{s}}_{\text{k}}\,\left(x\right)},K=4.$$ (6)

Window-level confidence and prediction entropy are expressed by Equation 7 and Equation 8, respectively:


$$C\,\left(x\right)=\max_{\text{c}}\text{p}\,\left(c|x\right),$$ (7)

$$E\left(x\right)=-\sum_{\text{c}=1}^{\text{K}}\text{p}\left(c|x\right)\log\text{[}p\left(c|x\right)+\varepsilon ].$$ (8)

A higher C indicates clearer judgment, whereas a higher E means flatter probability distribution, greater model hesitation, and larger individual differences in driver-state patterns.

### 2.4 Statistical analysis methods

As every participant experienced all four weather conditions, the data had a repeated-measures structure. We used the Friedman test for weather main effects, and Wilcoxon paired tests with false discovery rate (FDR) correction for multiple comparisons. For cross-modal associations, we used within-subject centered Spearman correlation. We first removed each participant’s own mean across the four weather conditions and subsequently computed correlation coefficients to eliminate individual baseline differences. The normality of the paired differences was assessed with Shapiro–Wilk tests; because several indicators deviated from normality, nonparametric tests were used throughout. We report 95% bootstrap confidence intervals (10,000 resamples) for key effects (Supplementary Table S1). A paired design with *N* = 30 detects dz = 0.53 at α = 0.05 and power = 0.80, below all headline effects. The cross-modal correlations reported in Section 3.5 were not corrected for multiple testing and are exploratory.

We expressed the change from clear to adverse weather via paired Cohen’s d_z_ as expressed by Equation 9:


$${\text{d}}_{z}=\frac{\bar{\text{d}}}{{\text{s}}_{\text{d}}},{\text{d}}_{\text{i}}={\text{x}}_{i,\mathrm{adverse}}-x_{i,\mathrm{clear}}.$$ (9)

The within-subject centered value is expressed by Equation 10:


$${\tilde{x}}_{i\,j}^{\left(k\right)}=x_{i\,j}^{\left(k\right)}-{\bar{x}}_{i}^{\left(k\right)}$$ (10)

## 3 Results

### 3.1 Subjective state analysis

Table 1 summarizes the descriptive statistics of NASA-TLX and KSS across the four tested weather conditions. The clear-weather scores remained relatively low. After entering adverse weather conditions, mental demand, temporal demand, effort, and frustration increased. The Friedman tests yielded significant weather main effects on KSS, NASA-TLX total, mental demand, temporal demand, effort, and frustration.

**TABLE 1:** Weather main effects of subjective indicators (Friedman test).

Indicator	Clear (M ± SD)	Rain (M ± SD)	Snow (M ± SD)	Fog (M ± SD)	x^2^	Kendall’s W	pFDR
NASA-TLX	27.36 ± 13.85	38.32 ± 17.14	40.89 ± 16.75	39.85 ± 15.73	29.40	0.327	< 0.001
KSS	2.00 ± 1.17	3.57 ± 1.38	3.43 ± 1.22	2.93 ± 1.39	38.04	0.423	< 0.001
Mental demand	9.18 ± 4.25	11.29 ± 4.59	11.93 ± 4.95	11.90 ± 4.52	18.52	0.206	0.001
Temporal demand	6.54 ± 4.66	8.75 ± 4.76	9.48 ± 4.76	8.91 ± 4.39	22.95	0.255	< 0.001
Effort	7.87 ± 4.61	10.61 ± 5.33	11.21 ± 4.97	10.65 ± 4.79	29.71	0.330	< 0.001
Frustration	3.76 ± 2.94	7.68 ± 4.40	8.28 ± 4.22	8.39 ± 4.52	32.62	0.362	< 0.001

**FIGURE 3:**
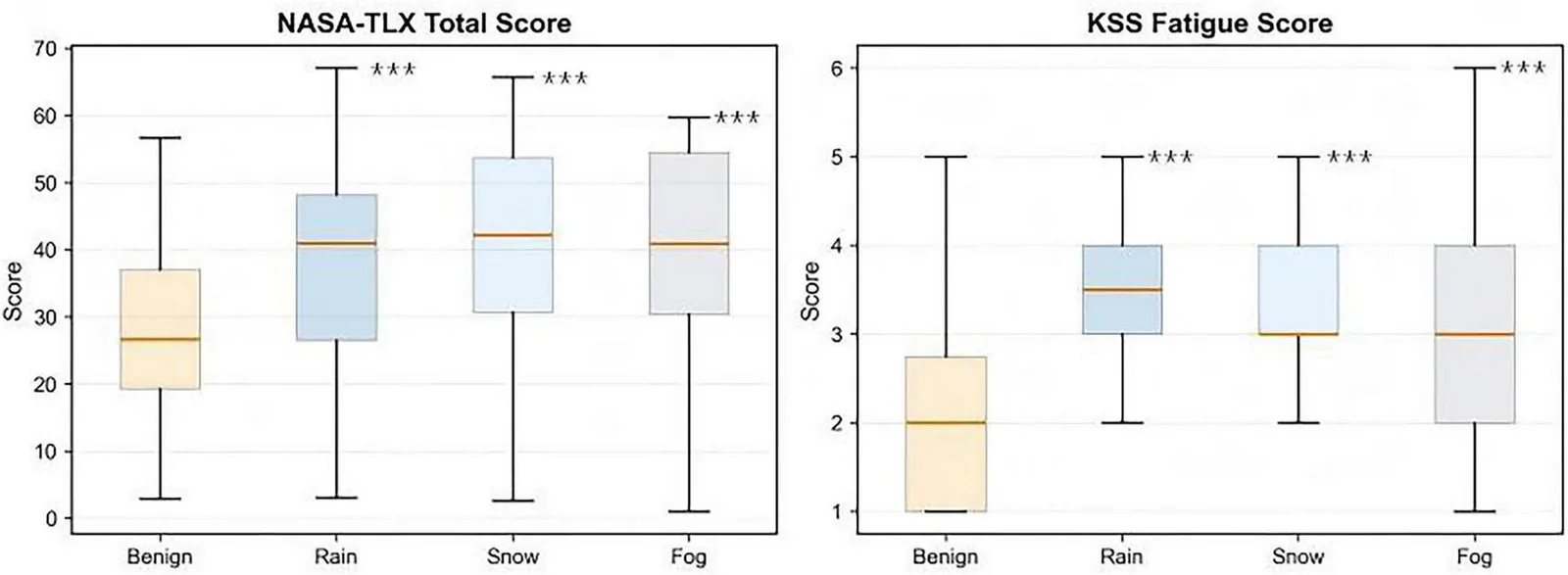
Subjective workload (NASA-TLX) and fatigue (KSS) across weather conditions (boxplots over 30 participants; asterisks indicate FDR-corrected contrasts vs. clear: *p_FDR < 0.05, **p_FDR < 0.01, ***p_FDR < 0.001).

Paired tests further showed that all three adverse weather types yielded scores significantly higher than that of the clear condition (Table 2 and Figure 3). Snow produced the largest increase in TLX, with a mean difference of 13.53 points and Cohen’s d_z_ = 1.29, indicating a large effect; rain and fog showed similar increases, with mean differences of 10.96 and 12.49 points and d_z_ = 0.96 and 1.26, respectively. Rain and snow led to particularly strong increases in KSS, both d_z_ > 1.0, approaching the threshold of “somewhat sleepy.” Overall, frustration increased more than mental demand, suggesting that negative emotional responses are likely more sensitive than increases in cognitive load.

**TABLE 2:** Pairwise comparisons of subjective indicators (adverse vs. clear).

Indicator	Rain vs. clear	Snow vs. clear	Fog vs. clear
NASA-TLX	△ = 10.96, d_z_ = 0.96, pFDR < 0.001	△ = 13.53, d_z_ = 1.29, pFDR < 0.001	△ = 12.49, d_z_ = 1.26, pFDR < 0.001
KSS	△ = 1.57, d_z_ = 1.09, pFDR < 0.001	△ = 1.43, d_z_ = 1.26, pFDR < 0.001	△ = 0.93, d_z_ = 0.76, pFDR = 0.001
Mental demand	△ = 2.10, d_z_ = 0.60, pFDR = 0.013	△ = 2.74, d_z_ = 0.85, pFDR = 0.002	△ = 2.71, d_z_ = 0.90, pFDR < 0.001
Temporal demand	△ = 2.21, d_z_ = 0.68, pFDR = 0.003	△ = 2.94, d_z_ = 0.96, pFDR < 0.001	△ = 2.37, d_z_ = 0.71, pFDR = 0.005
Effort	△ = 2.74, d_z_ = 0.68, pFDR = 0.004	△ = 3.34, d_z_ = 1.04, pFDR < 0.001	△ = 2.78, d_z_ = 1.02, pFDR < 0.001
Frustration	△ = 3.91, d_z_ = 1.06, pFDR < 0.001	△ = 4.52, d_z_ = 1.20, pFDR < 0.001	△ = 4.62, d_z_ = 1.35, pFDR < 0.001

These results indicate that all three adverse weather types significantly increased the drivers’ subjective load and fatigue, but the causes and accompanying features differed: snow imposed the heaviest task load, rain and snow produced more prominent fatigue effects, and negative emotional responses were sensitive across all three weather types.

### 3.2 Objective-indicator analysis

Before the entropy analyses, we reported CORF’s recognition performance: on out-of-fold predictions (6,456 windows), 91.9% accuracy and a Macro-F1 of 0.919, with per-class F1 from 0.898 (snow) to 0.942 (clear) and comparable calibration (Supplementary Table S2). Confusions occurred mainly between rain–snow and snow–fog pairs; snow was thus both the hardest state to recognize and the state with the highest response ambiguity.

Table 3 lists descriptive statistics for the EEG, ECG, and vehicle indicators across the four weather conditions. The mean heart rate exhibited a significant weather main effect (*p* = 0.015), with rain producing the largest increase. Regarding the EEG, C4 spectral entropy was higher under rain, snow, and fog than under clear weather; the Friedman test reached marginal significance (*p* = 0.067), and exploratory paired comparisons showed higher values under all three adverse weather types than under clear weather (Table 4). Regarding vehicle control, the speed mean dropped most strongly under fog, the speed standard deviation and RMS acceleration decreased under fog, and the absolute lateral offset remained high under rain, snow, and fog, with the Friedman test reaching marginal significance (*p* = 0.080). Prediction entropy showed a highly significant weather main effect (*p* < 0.001); the snow-versus-clear comparison exhibited a large effect, and the dual-entropy pathway analysis showed that the snow prediction entropy reached 0.755, the highest across the four weather types Table 5), indicating that the increase in system uncertainty under snow is statistically significant and practically meaningful.

**TABLE 3:** Weather main effects of key objective indicators (Friedman test).

Indicator	Clear (M ± SD)	Rain (M ± SD)	Snow (M ± SD)	Fog (M ± SD)	x^2^	Kendall’s W	pFDR
Speed mean	13.28 ± 1.90	12.56 ± 2.50	12.49 ± 2.70	11.57 ± 1.66	12.28	0.136	0.006**
Speed SD	0.51 ± 0.14	0.49 ± 0.25	0.56 ± 0.29	0.41 ± 0.15	11.96	0.133	0.008**
RMS acceleration	0.22 ± 0.07	0.23 ± 0.13	0.26 ± 0.15	0.18 ± 0.06	10.36	0.115	0.016*
Steering SD	0.0078 ± 0.0023	0.0066 ± 0.0038	0.0072 ± 0.0039	0.0056 ± 0.0037	11.24	0.125	0.010*
Lateral offset	19.75 ± 12.88	31.25 ± 18.54	29.62 ± 14.25	31.42 ± 17.93	6.76	0.075	0.080
HR mean	120.29 ± 26.92	132.62 ± 34.20	127.66 ± 33.06	123.43 ± 32.25	10.48	0.116	0.015*
C4 spectral Ent.	0.53 ± 0.12	0.57 ± 0.11	0.57 ± 0.11	0.56 ± 0.10	7.16	0.080	0.067
CORF Pred. Ent.	0.62 ± 0.13	0.70 ± 0.15	0.75 ± 0.09	0.73 ± 0.15	18.92	0.210	< 0.001***

Bold values indicate statistical significance after FDR correction (pFDR < 0.05).

*pFDR < 0.05,

**pFDR < 0.01,

***pFDR < 0.001.

**TABLE 4:** Pairwise comparisons of objective indicators (adverse vs. clear).

Indicator	Rain vs. clear	Snow vs. clear	Fog vs. clear
Speed mean	△ = −0.71, d_z_ = −0.30, pFDR=0.077	△ = −0.79, d_z_ = −0.28, pFDR=0.077	△ = −1.71, d_z_ = −0.81, pFDR<<0.001***
Speed SD	△ = −0.01, d_z_ = −0.06, pFDR=0.761	△ = 0.05, d_z_ = 0.23, pFDR=0.367	△ = −0.10, d_z_ = −0.64, pFDR=0.007**
RMS acceleration	△ = 0.01, d_z_ = 0.10, pFDR=0.641	△ = 0.04, d_z_ = 0.34, pFDR=0.138	△ = −0.04, d_z_ = −0.60, pFDR=0.012*
Steering SD	△ = −0.0013, d_z_ = −0.37, pFDR=0.151	△ = −0.0006, d_z_ = −0.17, pFDR=0.404	△ = −0.0023, d_z_ = −0.56, pFDR=0.008**
Lateral offset	△ = 11.51, d_z_ = 0.51, pFDR=0.047*	△ = 9.88, d_z_ = 0.61, pFDR=0.008**	△ = 11.68, d_z_ = 0.57, pFDR=0.016*
HR mean	△ = 12.32, d_z_ = 0.47, pFDR=0.046*	△ = 7.36, d_z_ = 0.34, pFDR=0.276	△ = 3.14, d_z_ = 0.16, pFDR=0.626
C4 spectral Ent.	△ = 0.04, d_z_0.47, pFDR=0.035*	△ = 0.05, d_z_ = 0.55, pFDR=0.008**	△ = 0.04, d_z_ = 0.45, pFDR=0.047*
CORF Pred. Ent.	△ = 0.08, d_z_ = 0.48, pFDR=0.017*	△ = 0.14, d_z_ = 1.21, pFDR<<0.001***	△ = 0.11, d_z_ = 0.67, pFDR = 0.006**

Bold values indicate statistical significance after FDR correction (pFDR < 0.05).

*pFDR < 0.05,

**pFDR < 0.01,

***pFDR < 0.001.

**TABLE 5:** Dual-entropy weather pathway values.

Weather	C4 SE mean	C4 SE SD	CORF PE mean	CORF PE SD
Clear	0.525	0.125	0.620	0.131
Rain	0.570	0.111	0.698	0.147
Snow	0.572	0.109	0.755	0.088
Fog	0.564	0.097	0.727	0.148

**FIGURE 4:**
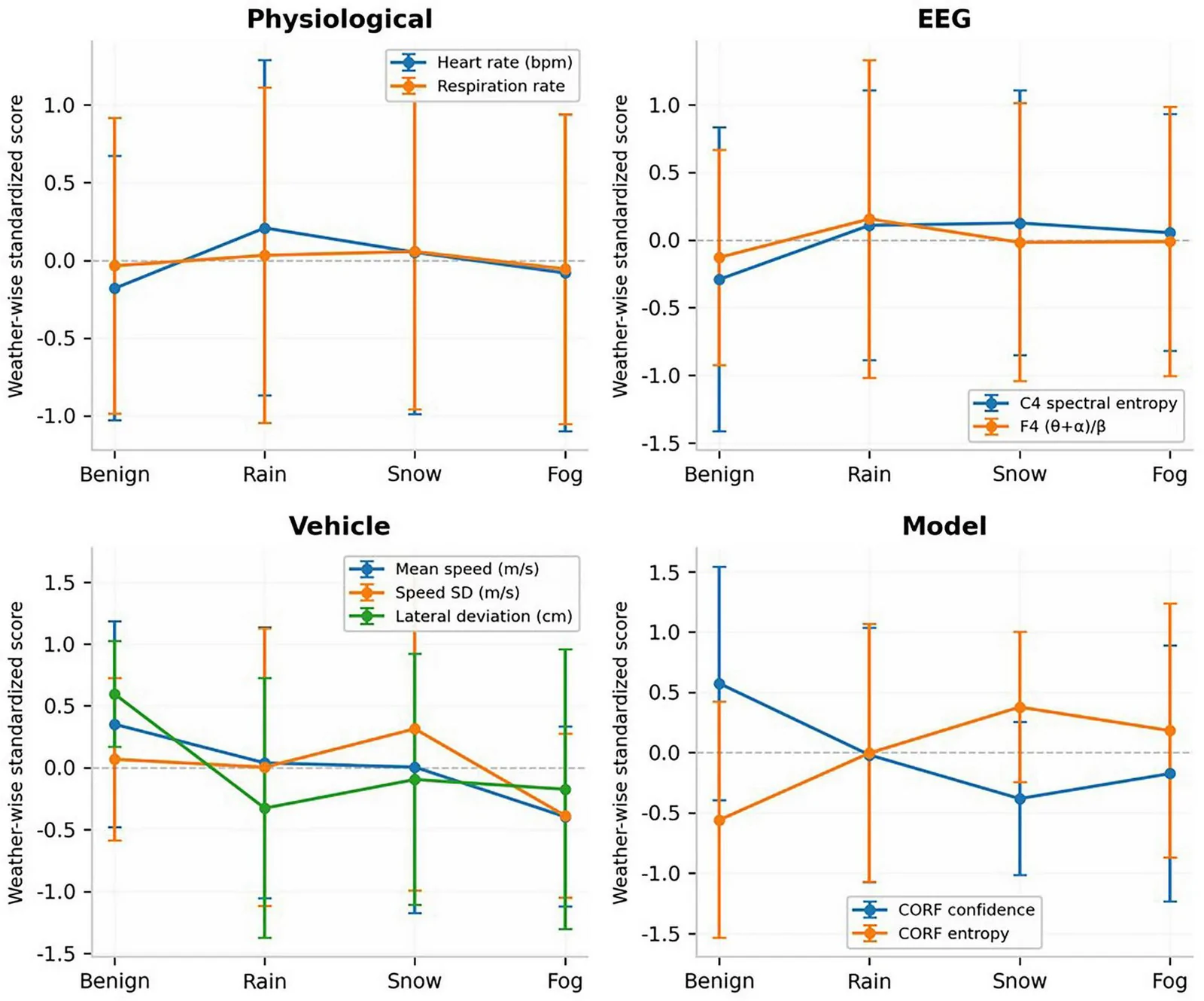
Multimodal response profiles across weather conditions (standardized scores by modality, M ± SD).

The paired comparisons further clarified the effect size and significance of these changes (Figure 4). For the rain condition, the mean heart rate was significantly higher than that in the clear weather condition, indicating adaptive autonomic regulation. Under snow conditions, the absolute lateral deviation reached an effect size of 0.61, one of the largest increases among the vehicle behavior indicators, indicating that snow most directly affects lane-keeping stability. Under fog conditions, the speed mean decreased significantly, but the speed standard deviation and RMS acceleration did not increase; instead, they exhibited a downward trend, indicating that drivers adopted a conservative strategy of active slowing and reduced operational fluctuation.

These selective changes in the objective indicators indicate that different weather types do not uniformly worsen all driver responses; they act at specific levels through different pathways. Rainfall primarily triggers cognitive processing and physiological arousal responses, whereas vehicle-operation fluctuations are actively suppressed. Snow simultaneously affects control stability and model-discrimination stability, with drivers’ coping strategies diverging markedly. Fog mainly alters speed-selection strategies, compensating for visual information shortages by reducing operational fluctuations.

**FIGURE 5:**
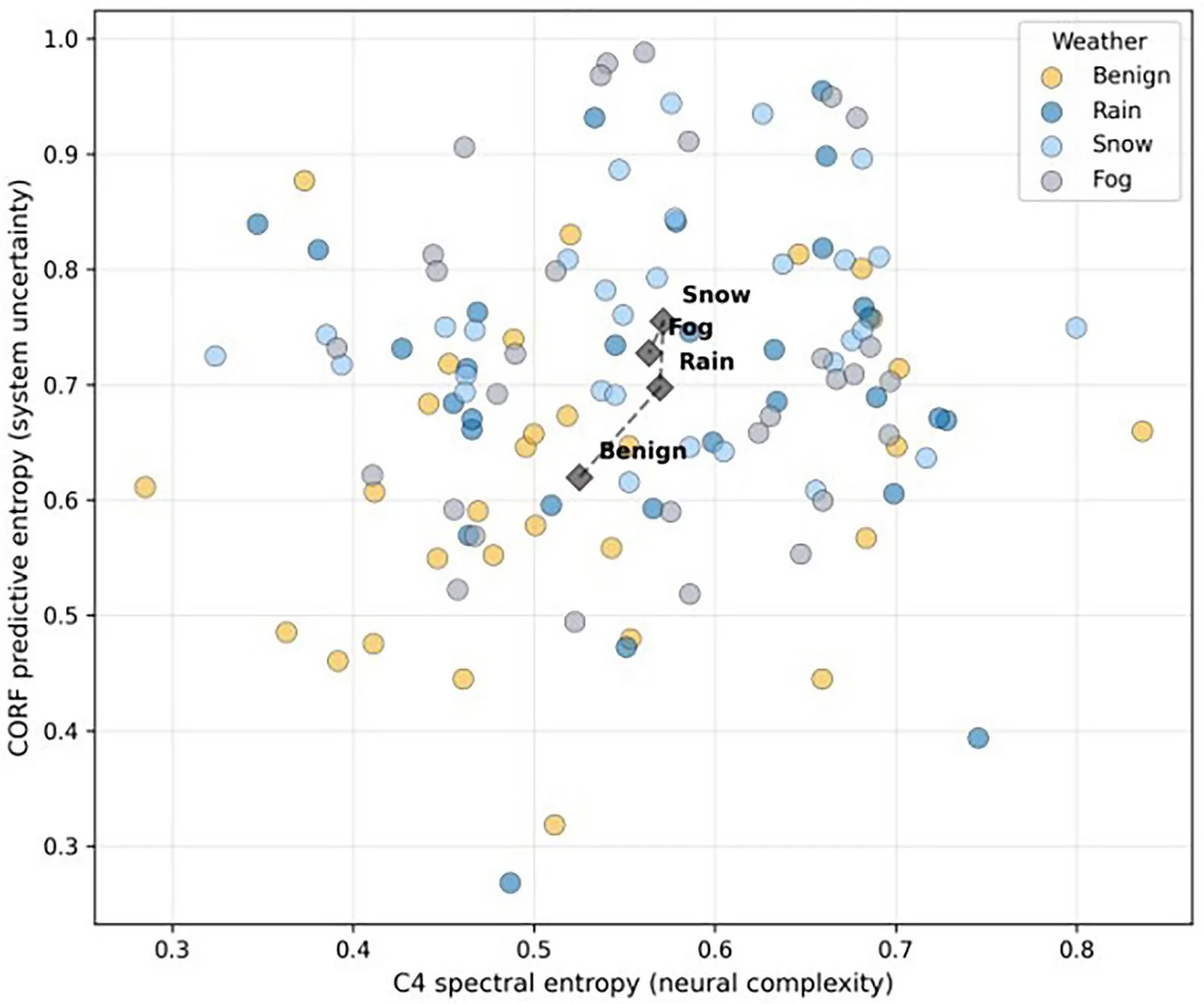
Dual-entropy weather pathways: C4 spectral entropy vs. CORF prediction entropy (120 participant × weather points).

To reveal how neural complexity and system uncertainty change together across weather types, we mapped the C4 spectral and CORF prediction entropies onto the same two-dimensional space. Figure 5 shows that the four weather types are distributed differentially along the neural-complexity versus system-uncertainty axis: clear sites at the lower left, with C4 spectral entropy of 0.525 and prediction entropy of 0.620; rain and fog shift toward the upper right, with prediction entropy increasing to 0.698 and 0.727, respectively; moreover, snow occupies the upper rightmost position, with C4 spectral entropy reaching 0.572 and prediction entropy reaching 0.755. This trajectory indicates that rain, snow, and fog do not possess different degrees of the same abnormal state; they occupy distinct positions in the information dynamics space as qualitatively different states. Snow showed simultaneous highs in both neural complexity and system uncertainty, fog was dominated by a prediction-entropy increase, and rain occurred in the intermediate transition zone.

To examine whether this entropy increase is specific to C4, spectral entropy was computed identically for all eight channels (Supplementary Table S3 and Supplementary Figure S1). The snow-related increase was FDR-significant at the six (among the eight) channels, following a centro-parietal gradient, indicating a distributed phenomenon rather than a single-channel artifact. Ranked by the mean effect size, C4 was placed second (among the eight channels) (mean *r* = 0.435, after Pz at 0.464). C4 was designated the primary channel a priori (Guo et al., 2015, 2016; pre-experimental screening); the all-channel results validate it as a representative site in this distributed pattern.

To rule out that this entropy pattern merely reflects classifier failure, the weather contrast was repeated using only the 5,933 correctly classified windows (Supplementary Table S4). The pattern persisted (snow vs. clear *r* = 0.749; fog *r* = 0.558; rain *r* = 0.426; all *p* < 0.02) with comparable calibration across weather types (expected calibration error = 0.153–0.170), whereas accuracy differed only modestly (89.7–93.8%) and entropy differed markedly (0.63 vs. 0.75 bits). Misclassified windows exhibited higher entropy than correct ones (1.084 vs. 0.656 bits); therefore, prediction entropy contains both model uncertainty and state ambiguity; our weather ranking rests on the correct-window control. Behavioral dispersion was also the largest under snow (across-participant standard deviation of speed variability: 0.287 vs. 0.144), consistent with divergent coping responses.

### 3.3 Composite-indicator analysis

Table 6 lists the M and SD values of all composite indicators across the four weather conditions, and Table 7 lists the pairwise comparisons relative to clear weather conditions. Subjective load increased significantly under all three adverse weather types, with snow exhibiting the largest increase. Operational fluctuations trended upward under snow conditions but did not reach significance; rain and fog also exhibited no significant changes. Conservative control significantly increased under fog conditions, reflecting a strengthened conservative driving strategy. Physiological arousal increased under rain and snow, with medium effect sizes relative to clear weather. The EEG response increased significantly in rain conditions, whereas the changes under snow and fog were smaller and did not reach significance. The prediction entropy significantly increased under snow conditions, indicating that the clear distinction of this weather’s multimodal response pattern by the model was the hardest.

**TABLE 6:** Weather main effects of multimodal composite pathways (Friedman test).

Pathway	Clear (M ± SD)	Rain (M ± SD)	Snow (M ± SD)	Fog (M ± SD)	x^2^	Kendall’s W	pFDR
Subjective load	−0.666 ± 0.687	0.281 ± 0.723	0.299 ± 0.731	0.086 ± 0.808	41.08	0.456	< 0.001***
Operational instab.	−0.042 ± 0.437	0.057 ± 0.717	0.213 ± 0.770	−0.229 ± 0.424	15.72	0.175	0.001**
Conservative ctrl.	−0.129 ± 0.482	−0.044 ± 1.056	−0.216 ± 1.125	0.389 ± 0.490	13.40	0.149	0.004**
Physio. arousal	−0.116 ± 0.676	0.144 ± 0.756	0.113 ± 0.810	−0.140 ± 0.686	12.68	0.141	0.005**
EEG response	−0.209 ± 0.655	0.133 ± 0.650	0.055 ± 0.567	0.022 ± 0.548	11.60	0.129	0.009**
Prediction entropy	−0.577 ± 0.944	−0.017 ± 1.060	0.396 ± 0.632	0.197 ± 1.068	18.92	0.210	< 0.001***

Bold values indicate statistical significance after FDR correction (pFDR < 0.05).

*pFDR < 0.05,

**pFDR < 0.01,

***pFDR < 0.001.

**TABLE 7:** Pairwise comparisons of composite pathways (adverse vs. clear).

Pathway	Rain vs. clear	Snow vs. clear	Fog vs. clear
Subjective load	△ = 0.95, d_z_ = 1.33, pFDR<<0.001***	△ = 0.97, d_z_ 1.45, pFDR<<0.001***	△ = 0.75, d_z_ 1.12, pFDR<<0.001***
Operational instab.	△ = 0.10, d_z_ = 0.16, pFDR=0.440	△ = 0.26, d_z_0.38, pFDR=0.115	△ = −0.19, d_z_ = −0.36, pFDR=0.109
Conservative ctrl.	△ = 0.09, d_z_ = 0.09, pFDR=0.612	△ = −0.09, d_z_−0.09, pFDR=0.612	△ = 0.52, d_z_ = 0.88, pFDR<<0.001***
Physio. arousal	△ = 0.26, d_z_0.45, pFDR=0.074	△ = 0.23, d_z_ = 0.31, pFDR=0.179	△ = −0.02, d_z_ = −0.04, pFDR=0.761
EEG response	△ = 0.34, d_z_ = 0.81, pFDR<<0.001***	△ = 0.26, d_z_ = 0.62, pFDR=0.003**	△ = 0.23, d_z_ = 0.53, pFDR=0.013*
Prediction entropy	△ = 0.56, d_z_0.48, pFDR=0.017*	△ = 0.97, d_z_ = 1.21, pFDR<<0.001***	△ = 0.77, d_z_ = 0.67, pFDR=0.006**

Bold values indicate statistical significance after FDR correction (pFDR < 0.05).

*pFDR < 0.05,

**pFDR < 0.01,

***pFDR < 0.001.

The composite indicators further revealed the dominant response mechanisms under the three adverse weather conditions. Rainfall is primarily driven by cognitive and physiological tension. The EEG response significantly increased with a large effect size, physiological arousal exhibited an upward trend but did not reach significance, and operational fluctuations exhibited no significant change. These findings show that rain’s environmental stimuli primarily act on drivers’ cognitive processing and autonomic regulation, whereas vehicular operation fluctuations are actively suppressed, forming a “cognitive tension–behavioral stability” pattern. Snow is characterized by jointly aggravated state-boundary blurring and system uncertainty. The prediction-entropy pathway increased significantly with a large effect size; however, neither operational fluctuation nor conservative control reached significance. Snow simultaneously reduces visibility, lowers road adhesion, and imposes cold temperature stress, and these stressors combine to drive coping strategies apart; thus, data dispersion within the same weather grows, and category boundaries become blurred. Fog was dominated by conservative compensation; conservative control increased significantly with a large effect size, operational fluctuation exhibited no significant change, and the prediction-entropy pathway increased significantly. This indicates that the core problem of fog is visual-information restriction; drivers actively adopt the conservative strategies of slowing down and reducing longitudinal fluctuation, using operational caution to compensate for visual-information shortage, forming a “risk perception–conservative execution” pattern.

**FIGURE 6:**
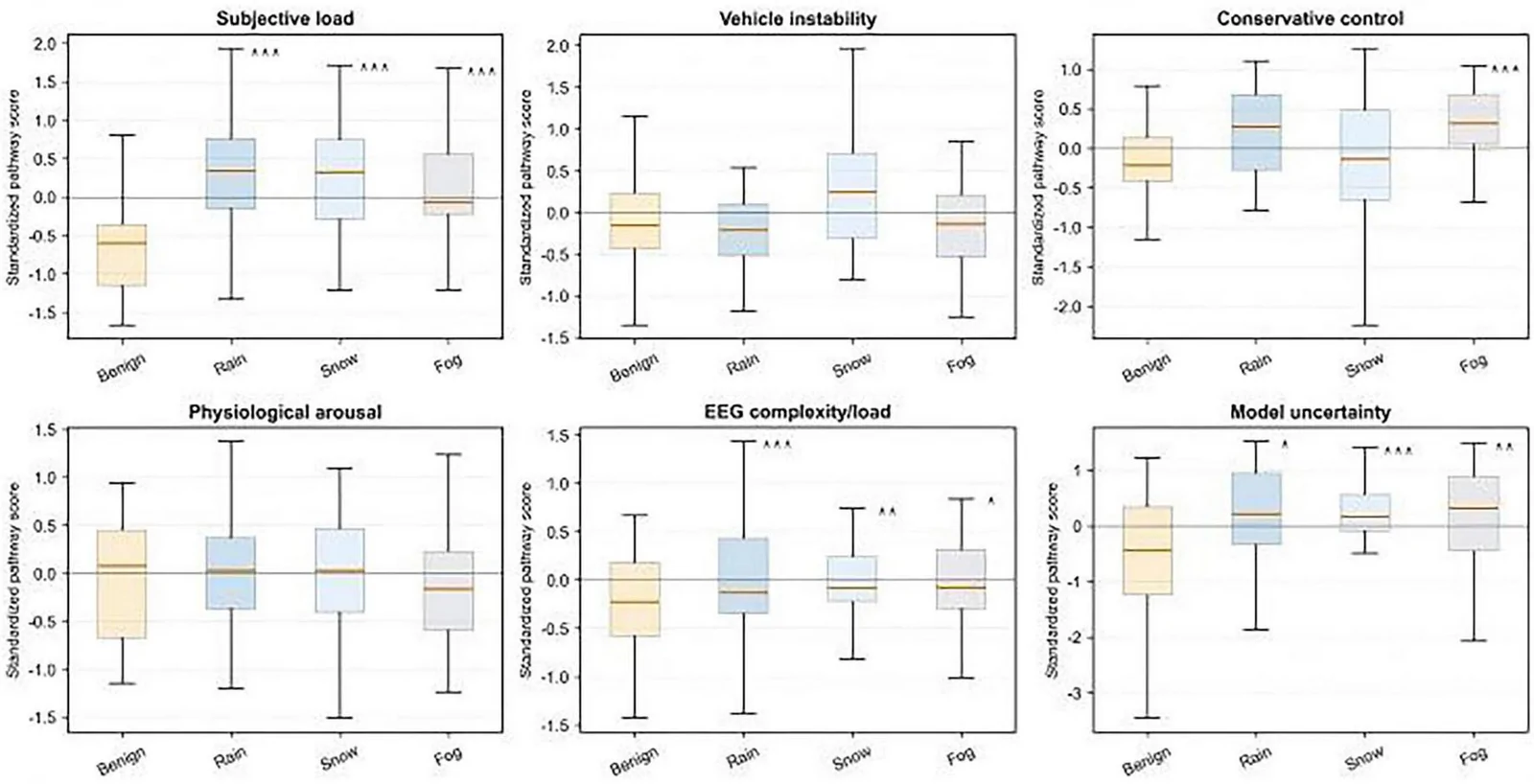
Weather-specific multimodal pathway scores (boxplots over the 120 participant × weather points; *p_FDR < 0.05, **p_FDR < 0.01, ***p_FDR < 0.001 vs. clear).

**FIGURE 7:**
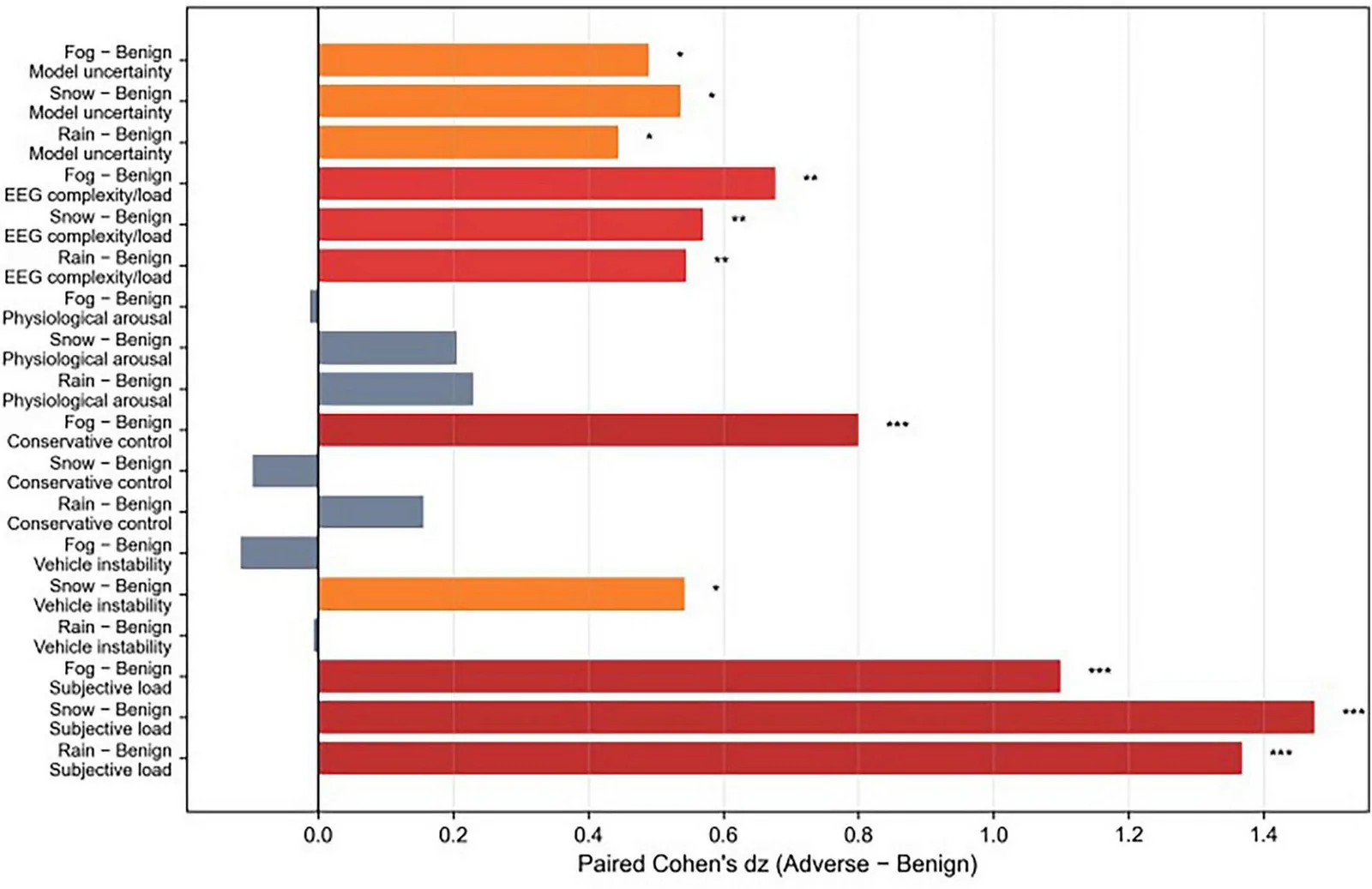
Effect sizes (paired Cohen’s dz) of pathway changes relative to the clear condition (*p_FDR < 0.05, **p_FDR < 0.01, ***p_FDR < 0.001).

**FIGURE 8:**
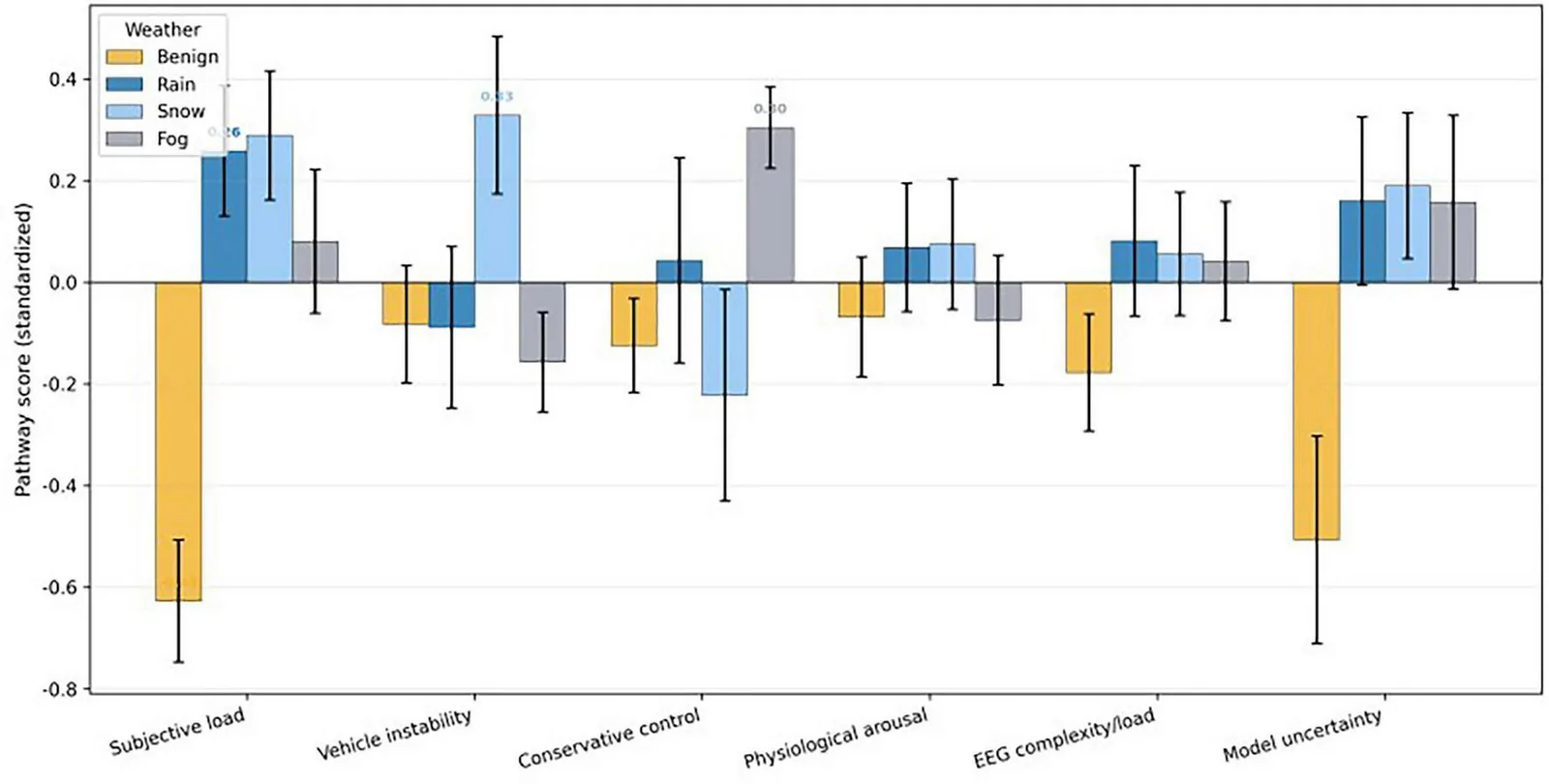
Weather-specific multimodal pathway comparison (standardized pathway scores, M ± SEM).

Figures 6–8 depict these differentiated patterns through boxplots, effect-size forest plots, and grouped bar charts. Rainfall formed a clear peak in the EEG response dimension, snow stood out most prominently in the prediction entropy dimension, and fog yielded the strongest performance in the conservative control dimension.

### 3.4 Multimodal-indicator association analysis

To quantify the information structure across the four modalities, we computed within-modality and cross-modality mean Spearman correlations. The averaged within-modality correlation value was 0.368, reflecting information redundancy among indicators in the same dimension. The averaged cross-modality correlation was 0.184, reflecting information complementarity across different dimensions. The difference of 0.184 indicates that different modalities contain non-overlapping system information. Table 8 lists the specific values. The within-modality correlation incorporated the mean absolute value of all pairwise combinations of raw indicators within each modality: one pair for subjective load, three for cardiac response, one for EEG activity, and six for vehicle control, amounting to overall 11 pairs. The cross-modality correlation incorporated the mean absolute value of all pairwise combinations of the four composite modality scores for overall six pairs.

**TABLE 8:** Quantification of information complementarity.

Metric	Value
Intra-modal redundancy	0.368
Inter-modal complementarity	0.184
Difference	0.184
Intra-modal pairs (n)	11
Inter-modal pairs (n)	6

**FIGURE 9:**
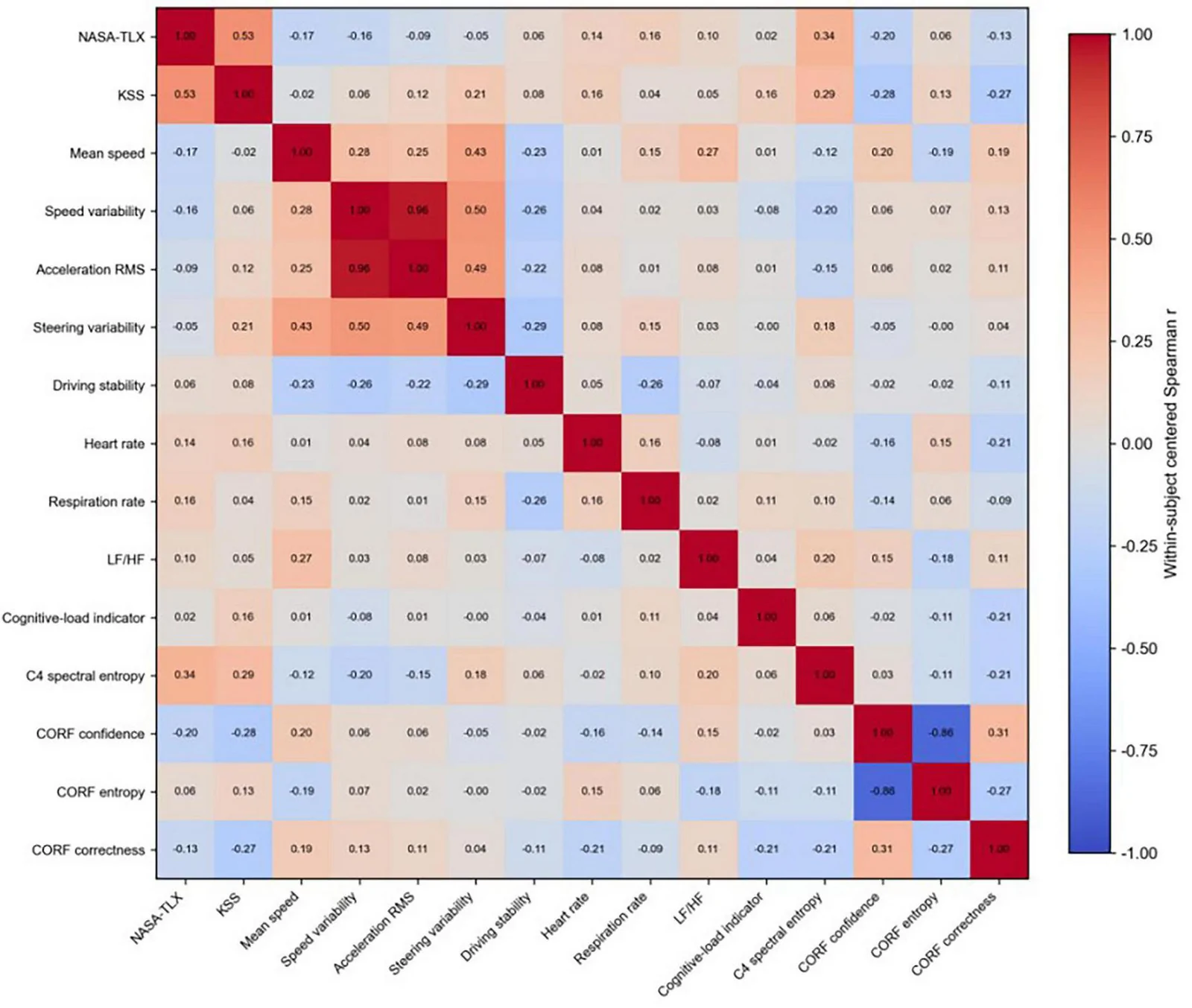
Within-subject multimodal correlation matrix (Spearman r after within-subject centering).

**FIGURE 10:**
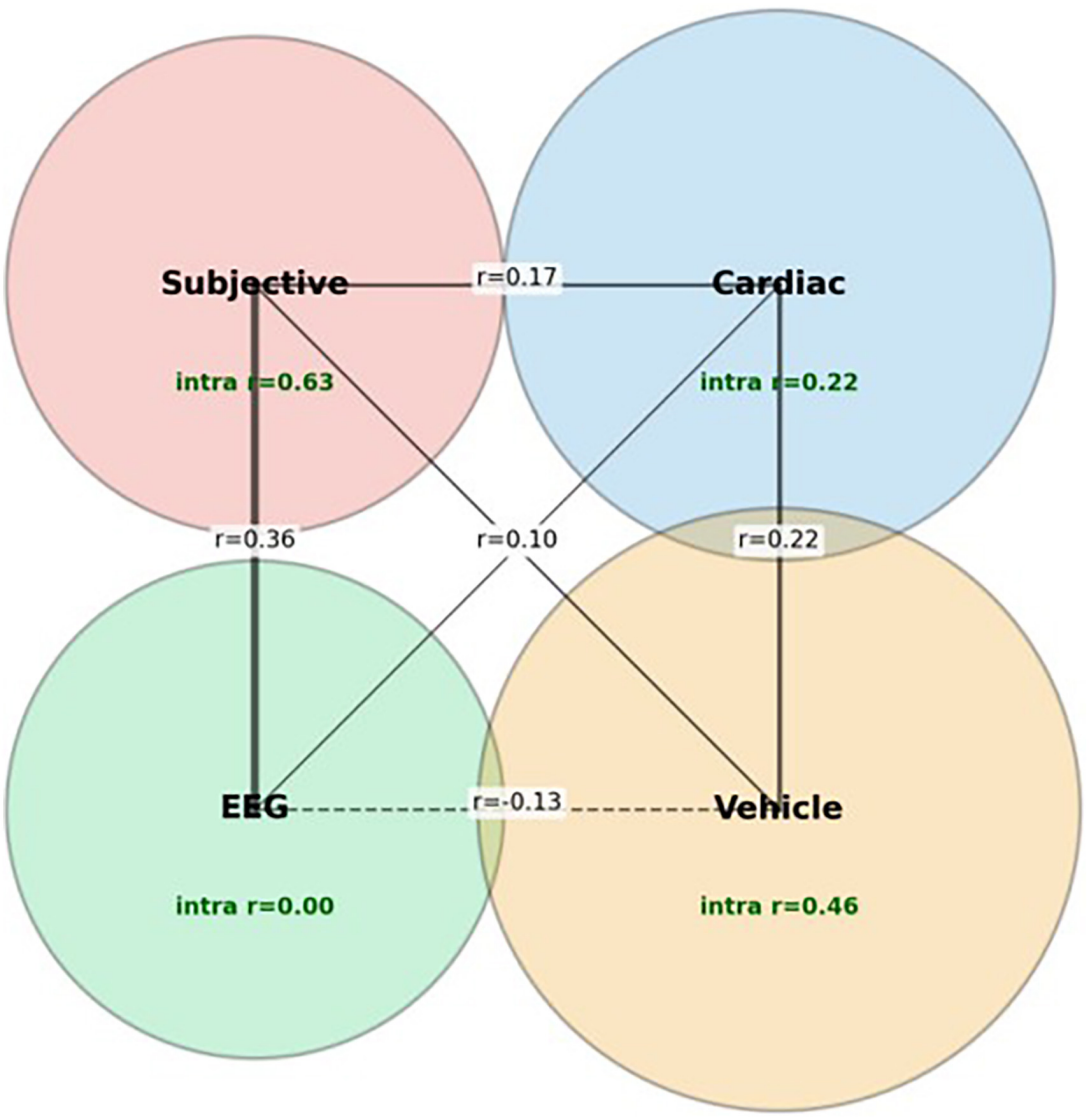
Information-complementarity structure: intra-modality redundancy vs. inter-modality complementarity across the four modalities.

The correlation matrix is shown in Figure 9. Key within- and cross-modality correlations are listed in Table 9. Figure 10 depicts this structure as a network. The four nodes—subjective load, cardiac response, EEG activity, and vehicle control—show moderate internal connections, with within-modality correlations ranging from 0.00 to 0.63; internode links are sparse, with cross-modality coefficients ranging from 0.10 to 0.63. The within-modality correlation of the EEG activity is almost zero because the F4 channel’s (θ+α)/β ratio and C4 channel’s spectral entropy reflect two independent dimensions—spectral ratio vs. spectral-entropy distribution—rather than measurement error. This “strong inside, weak outside” structure confirms that different facets of the driving state contain non-overlapping system information, and no single modality suffices for a complete characterization.

**TABLE 9:** Key correlations after within-subject centering.

Variable 1	Variable 2	r	P
NASA-TLX total	KSS	0.586	< 0.001
Speed SD	RMS acceleration	0.978	< 0.001
RMS acceleration	Steering SD	0.511	< 0.001
CORF confidence	CORF prediction Ent.	−0.894	< 0.001
CORF confidence	CORF accuracy	0.345	< 0.001
CORF prediction Ent.	CORF accuracy	−0.324	< 0.001
NASA-TLX total	HR mean	0.225	0.013
NASA-TLX total	CORF prediction Ent.	0.092	0.318
NASA-TLX total	C4 spectral Ent.	0.411	< 0.001

**FIGURE 11:**
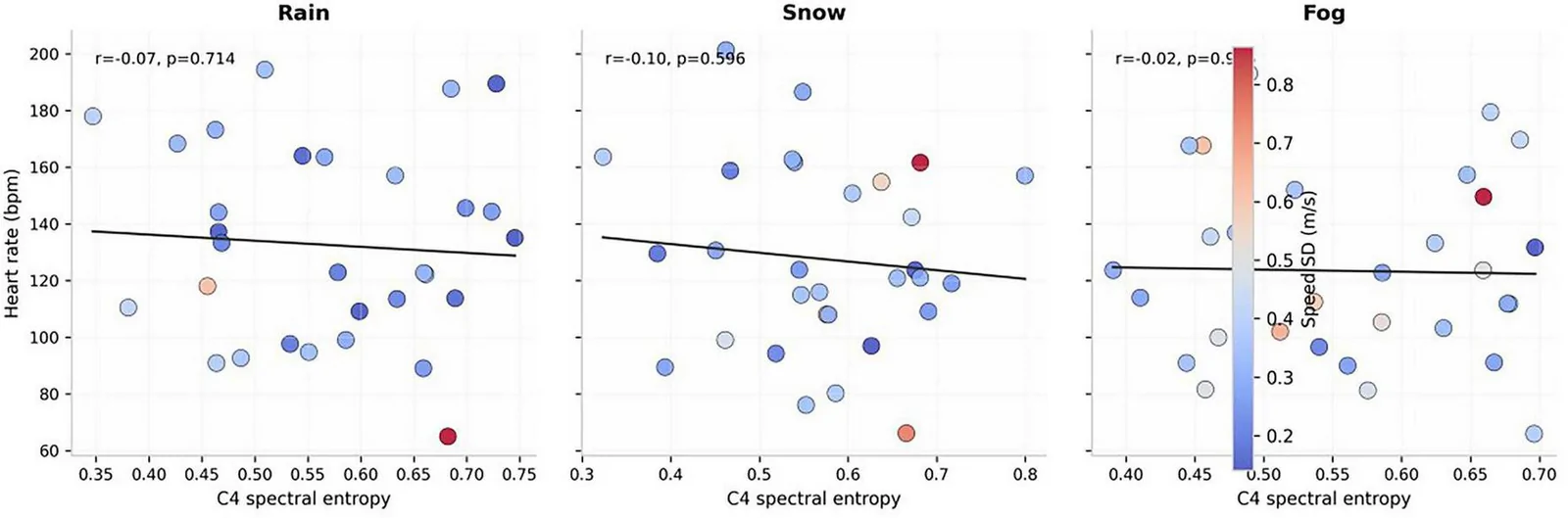
EEG–physiology–vehicle linkage across adverse weather (C4 spectral entropy vs. heart rate; color indicates speed SD; panels: rain, snow, fog).

Figure 11 shows the weather-specific scatter plots of the EEG, ECG, and vehicle indicators. The three subplots correspond to rain, snow, and fog; the horizontal axis is the C4 spectral entropy, vertical axis is the M of the heart rate, and point color indicates the SD of speed. In all three weather types, the trend line between EEG and heart rate was nearly horizontal, with non-significant correlation coefficients, visually corroborating the weak cross-modality conclusion. Moreover, in the snow subplot, point colors were more dispersed, indicating larger individual vehicular fluctuation differences under these weather conditions.

### 3.5 Nonlinear coupling and information gain

The analysis in section 3.4 shows that different modalities form an information-complementary structure, with the cross-modality correlation being substantially lower than the within-modality correlation, indicating that driving-state multidimensional responses may not be captured by simple linear combinations. Linear discriminant analysis (LDA) was used as a benchmark to test whether the driving state can be summarized using simple linear rules. The results showed that the LDA four-class balanced accuracy dropped to 0.375 near the random level, indicating that the rain, snow, and fog response patterns were not linearly separable in the feature space. To examine this nonlinear feature, we adopted a distance correlation analysis to quantify the nonlinear components in the cross-modal associations.

A distance correlation analysis quantified this nonlinearity. Table 10 lists the Spearman and distance correlation coefficients for the key cross-modal associations. The Spearman correlations in the table only describe the trend direction and were not corrected for multiple comparisons. Nonlinear gain, defined as the difference between distance correlation and Spearman correlation, does not depend on significance thresholds and counts as exploratory evidence. Key pairings, such as the EEG–subjective load, EEG–cardiac, and prediction-entropy–subjective load, showed positive nonlinear gains, with a mean of 0.073. Although the effect size was modest, the consistent direction indicates that cross-modal coupling contains nonlinear components that traditional linear discriminant models cannot fully explain. This finding corroborates the LDA result, showing that the true structure of the driving state involves multidimensional complex associations, and information entropy, as a model-free measure, provides a more effective description of the system’s nonlinear uncertainty.

**TABLE 10:** Nonlinear coupling test (distance correlation).

Variable X	Variable Y	Spearman r	Spearman p	dCor	Nonlinear△
C4 spectral Ent.	NASA-TLX total	0.201	0.028	0.237	0.037
C4 spectral Ent.	LF/HF	0.125	0.174	0.165	0.040
C4 spectral Ent.	HR mean	−0.056	0.542	0.139	0.083
CORF prediction Ent.	LF/HF	0.044	0.635	0.157	0.113
CORF prediction Ent.	NASA-TLX Total	0.181	0.048	0.244	0.064
CORF prediction Ent.	Speed SD	−0.041	0.653	0.145	0.103
Mean nonlinear gain	−	−	−‘	−	0.073

We constructed linear regression models with the CORF prediction entropy as the dependent variable, each modality’s composite indicator as the independent variable, and R2 as a measure of the modality’s explanatory power for system uncertainty. Table 11 indicates that the vehicle control had the highest explanatory power for the CORF prediction entropy, with R2 = 0.125, whereas for EEG activity, R2 = 0.052; for subjective load, R2 = 0.045; and for cardiac response, R2 = 0.006. However, the four-modality joint explanatory power reached 0.192, which was significantly higher than that of any single modality and close to the sum of the four single-modality R2 values (0.228). This difference remains within the expected range after incorporating cross-modality collinearity. This finding directly confirms the necessity of multimodal fusion in the information-complementary structure. Although vehicle control is the main outward manifestation of uncertainty, EEG activity and subjective load provide irreplaceable independent information.

**TABLE 11:** Information gain (R2 of CORF prediction entropy).

Modality	Indicator	R2	n
Subjective load	KSS + TLX total	0.045	120
ECG response	SDNN + HR + LF/HF	0.006	120
EEG activity	F4(θ+α)/β + C4 spectral Ent.	0.052	120
Vehicle control	Speed SD + offset + steering + RMS	0.125	120
Four-modality joint	All indicators	0.192	120

### 3.6 Individual differences and response typology

All the aforementioned analyses are based on group averages, yet driver response patterns under adverse weather conditions may exhibit substantial individual differences. Because median splits reduce statistical power and impose artificial group boundaries, driver heterogeneity was characterized with data-driven clustering (k-means with silhouette-based selection of the cluster number with Ward hierarchical clustering as a complement). The analysis is exploratory, revealing directional features rather than universal classification standards.

**FIGURE 12:**
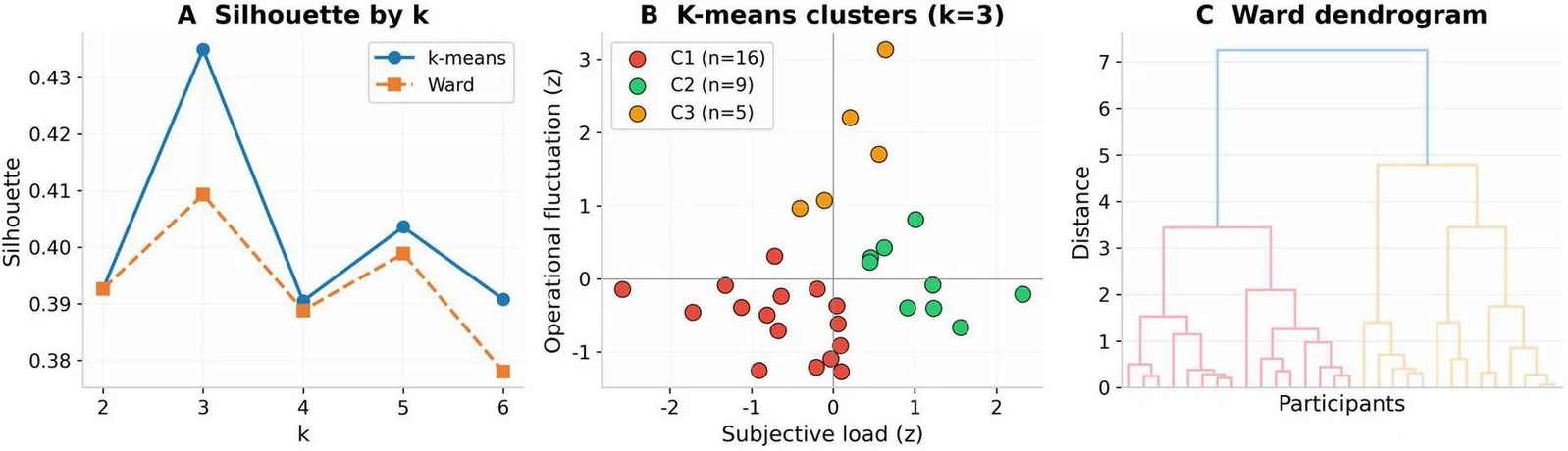
Exploratory driver typology. **(A)** Silhouette coefficients by k. **(B)** k-means clusters (*k* = 3) on standardized subjective load and operational fluctuation. **(C)** Ward dendrogram.

These descriptive statistics showed that snow yielded the highest system uncertainty across the four weather types, as reflected by the CORF prediction entropy and composite prediction-entropy pathways. The clustering of the 30 drivers further revealed divergent coping strategies under snow. We clustered each driver’s mean adverse-weather response along two dimensions—subjective load (NASA-TLX + KSS) and operational fluctuation (speed SD, RMS acceleration, steering SD, absolute lateral offset; all standardized). Silhouette analysis favored *k* = 3 (0.435, exceeding *k* = 2, 4, 5, 6; Ward clustering agreed; Figure 12). Table 12 lists the three clusters, and Supplementary Table S5 summarizes the per-participant labels.

**TABLE 12:** Three-cluster driver typology (k-means, *k* = 3).

Response type	n	%	Description
Strained-reactive	5	16.7%	High subjective load (TLX ≈ 43) with the largest control fluctuation and C4 spectral entropy
Stable	16	53.3%	Lowest subjective load and control fluctuation
Tense-compensating	9	30.0%	Highest subjective load (TLX ≈ 52; KSS ≈ 4.2) but only moderate fluctuation—tension managed through stable control

The 30 drivers fell into three response groups: a strained-reactive group (*n* = 5), combining high subjective load (NASA-TLX ≈ 43) with the largest control fluctuation and highest C4 spectral entropy; a stable group (*n* = 16), with the lowest load and fluctuation; and a tense-compensating group (*n* = 9), with the highest load (NASA-TLX ≈ 52; KSS ≈ 4.2) but only moderate fluctuation, suggesting that tension is managed through stable control. The three-cluster solution was stable across 500 half-sample iterations (median adjusted Rand index = 0.755). An initial median-split four-type description agreed only weakly with the clusters [adjusted Rand index (ARI) = 0.29] and was abandoned.

These individual differences do not have random noise characteristics; they represent stable behavioral pattern differentiation and are the key source of the blurred state boundaries of snow. The tense-compensating group may maintain operational stability through experience-based compensation, whereas the strained-reactive group may benefit mostly from early, graded warnings. Given the small subgroup sizes, these types are hypothesis-generating and require validation in larger samples (Gace et al., 2021; Tselentis and Papadimitriou, 2023). Overall, the results support the proposed hypothesis: rain, snow, and fog elicit qualitatively different multimodal response patterns, and the two entropy metrics capture complementary aspects of these patterns. The research objectives—quantifying weather-specific response pathways and characterizing individual differences—were therefore met.

## 4 Discussion

Leveraging multimodal sensor data from a driving simulator experiment with 30 licensed drivers across clear, rain, snow, and fog conditions, this study employed C4 spectral entropy and CORF prediction entropy as information-theoretic tools to reveal the coupling mechanism among subjective load, cardiac response, EEG activity, and vehicle control under adverse weather conditions from a system-uncertainty perspective.

Rainfall, snow, and fog significantly increased the subjective load, yet the dominant pathways differed fundamentally. Rain was dominated by cognitive-load increase; EEG response increased significantly with a large effect size, reflecting more complex EEG activity and moderate system-uncertainty growth; and drivers maintained operational stability through higher cognitive engagement, forming a “cognitive tension-behavioral stability” pattern. Snow showed synchronized increases in neural complexity and system uncertainty, prediction entropy reached its highest level across the four weather types, the prediction-entropy pathway increased significantly with a large effect size, drivers adopted highly divergent coping strategies, and state-boundary blurring intensified, making the model the most hesitant and uncertain. This finding extends the observation by Kamti et al. (2023), who reported large individual differences under rain and fog, by showing that snow induces the largest individual divergence, likely due to the combined stressors of low visibility, slippery surfaces, and cold temperature. Similarly, Zhang et al. (2025) showed that uncertainty-weighted fusion of EEG and EOG improves fatigue detection robustness; our results further demonstrate that this type of uncertainty information can also reveal how different adverse weather types affect driver-state stability, with snow producing the greatest system uncertainty. Fog was characterized by conservative compensation under visual restriction conditions. Prediction entropy increased, yet C4’s spectral entropy increased only slightly. Conservative control increased significantly with a large effect size, and drivers actively decelerated to suppress operational-uncertainty growth, with speed SD and RMS acceleration actually decreasing, forming a “risk perception-conservative execution” pattern. Unlike Chang et al. (2019), who found that fog led to increased speed dispersion, our results show that drivers under fog conditions actively reduced speed and speed variability, suggesting a conservative compensation strategy rather than unstable control. These three pathways show that rain, snow, and fog are not different degrees of the same abnormal state; they occupy distinct positions in the information-dynamics space as qualitatively different states: snow shows the highest system uncertainty and largest individual divergence; fog is dominated by a prediction-entropy increase with controlled behavioral entropy; and rain occupies the intermediate transition zone. The observed entropy increases are consistent with entropy-based accounts of workload and fatigue in EEG and HRV, which link rising signal complexity to sustained cognitive demand (Perrey, 2025; Kao et al., 2025; Lin et al., 2018).

The four modalities have an information-complementary structure. The within-modality mean Spearman correlation was substantially higher than the cross-modality mean, and the positive difference indicated that the different modalities carry non-overlapping system information. The within-modality correlation of EEG activity is near zero because the F4 channel’s (θ+α)/β ratio and C4 channel’s spectral entropy reflect two independent dimensions—spectral ratio versus spectral-entropy distribution—rather than measurement error. The explanatory power of each modality for prediction entropy also differed markedly: vehicle control was the highest, EEG activity followed, subjective load was the next, and the cardiac response was the lowest. The four-modality joint explanatory power was higher than that of any single modality and approached the sum of the individual contributions; this difference falls within the expected range after accounting for cross-modality collinearity. This confirms that the driving state cannot be linearly depicted by a single indicator, and the necessity of multimodal fusion lies not only in improving the recognition accuracy but also in the irreplaceable independent information provided by each modality. We distinguish the data-side ambiguity of the driver–vehicle system from classifier reliability (Huseljic et al., 2020); the correct-window control and calibration checks in section 3.2 support the former assertion.

To test whether the driving state can be captured by simple linear rules, we used LDA as a benchmark; its four-class balanced accuracy was near a random level, indicating that the response patterns to rain, snow, and fog are not linearly separable in the feature space. Distance correlation analysis further showed that the key cross-modal associations had positive nonlinear gains, and pairings such as EEG–subjective load and prediction-entropy–subjective load showed consistent positive gains, indicating that cross-modal coupling contains nonlinear components. As argued by Perrey (2025), conventional linear EEG analysis fails to capture the inherent complex dynamics of neural signals, whereas entropy—a model-free measure—provides a more effective description of these types of nonlinear fluctuations. Traditional variance or linear models assume additivity and fixed relationship forms, making them unable to describe the interaction of multidimensional heterogeneous information. Information entropy, as a model-free measure that does not depend on distributional assumptions or linear premises, captures the complexity and uncertainty of this system more accurately. This study extended spectral entropy and prediction entropy from model evaluation metrics to tools for analyzing system complexity, providing an information-theoretic uncertainty quantification framework for driver-state monitoring under adverse weather conditions. These findings underscore the necessity of multimodal sensor fusion for capturing the complex, nonlinear nature of driver states under adverse weather and highlight the value of entropy-based measures as interpretable, model-free descriptors in sensor-based monitoring systems.

The drivers demonstrated distinct individual differences. Exploratory clustering grouped the 30 participants into three response types (strained-reactive, stable, and tense-compensating), recovered with a median ARI of 0.755 across half-sample iterations. This differentiation is not random noise but the key source of the blurred state boundaries of snow, which produced the highest system uncertainty across the four weather types and most divergent coping strategies, indicating the largest individual differences in system uncertainty. Individual differentiation of this kind is consistent with previous reports of stable, driver-specific response patterns under challenging conditions (Gace et al., 2021; Tselentis and Papadimitriou, 2023). In contrast, studies relying on group-average indicators have generally reported uniform shifts in driving behavior under adverse weather; the present results indicate that such averages can mask divergent individual coping strategies, particularly under snow. Tense-compensating drivers may maintain operational stability through experience-based compensation under snow conditions, whereas strained-reactive drivers may benefit mostly from early, graded warnings. Driver-assistance systems should not rely entirely on group-average models; they require personalized calibration according to the drivers; basic response types.

Several limitations should be noted. First, the experiment was conducted in a driving simulator: although it allowed precise control of conditions, it lacked the constraints imposed by actual traffic conditions. Simulator validation studies support relative rather than absolute validity (Meuleners and Fraser, 2015; Pawar et al., 2022); our findings are weather-relative response patterns requiring naturalistic replication. Second, the sample (*N* = 30) is limited owing to the pilot scale, constrained by equipment and recruitment; the repeated-measures design and sensitivity analysis (minimum detectable dz = 0.53, below all headline effects) support the weather-level conclusions, but subgroup and nonlinear findings are exploratory. Third, the eight-channel EEG montage is the full capacity of the available wireless system; coverage is sparse, and the spectral-entropy effect is distributed over centro-parietal sites rather than being confined to C4. Fourth, prediction entropy is model-dependent: absolute values may shift with other classifiers or calibration methods, although out-of-fold estimation, comparable calibration, and the correct-window control bound this dependence; the 50% window overlap may allow limited leakage between adjacent windows. Finally, HRV analysis was restricted to SDNN and LF/HF (based on the 10-s windows); window averages were used rather than temporal dynamics, and the three driver types are exploratory and require on-road validation in larger samples.

## 5 Conclusion

This study compared the multimodal responses of 30 drivers across clear, rain, snow, and fog conditions in a within-subject simulator experiment, using the two complementary entropy metrics. The three adverse weather types yielded distinct response patterns: rain, a cognitive-tension pattern; snow jointly elevated neural complexity and state-boundary ambiguity with divergent coping responses; and fog, a conservative-compensation pattern with reduced speed. The four modalities showed an information-complementary structure, and exploratory clustering suggested three driver response groups. As the evidence is correlational and simulator-based, these are weather-relative associations and not established causal mechanisms. Nevertheless, the dual-entropy metrics provided a quantifiable basis for weather-aware driver-state monitoring and a graded warning design. The prominent inference is that adverse weather reshapes the driver–vehicle system along weather-specific pathways rather than simply degrading performance. Theoretically, the dual-entropy framework provides a reproducible measure of state-boundary ambiguity; practically, it supports personalized, weather-adaptive warning calibration. Future work should validate these weather-relative patterns in naturalistic on-road driving with larger samples, denser EEG coverage, and longer HRV windows, and should test whether entropy-based graded warnings improve safety outcomes.

## Data Availability

The original contributions presented in the study are included in the article/Supplementary material, further inquiries can be directed to the corresponding author.
