# Supplementary material for: Multimodal sensor-based response mechanisms of drivers under adverse weather conditions using EEG spectral entropy and prediction entropy
Source: Front Neurosci. 2026 Sep 9;20:1919198. doi: 10.3389/fnins.2026.1919198 (PMC13597921; doi:10.3389/fnins.2026.1919198)
Supplement: Supplementary file 1 [file Supplementary_file_1.zip › Supplementary_Material/补充材料_Supplementary_Material.docx]

# Supplementary Material

**Multimodal Sensor-Based Response Mechanisms of Drivers Under Adverse Weather Conditions Using EEG Spectral Entropy and Prediction Entropy**

Yi Tian, Jianping Hu, Hao Ding, Binhe Yang, Jialin Hu, Yuting Liu, Yu Ding

## Supplementary Figures

Figure S1. All-channel spectral entropy by channel and weather condition. (A) Per-channel effect sizes (rank-biserial r) of the adverse-weather vs. clear contrasts; (B) group means of spectral entropy across the four weather conditions for all eight EEG channels. The snow-related increase is FDR-significant at six of eight channels and follows a centro-parietal gradient.

## Supplementary Tables

## **Table S1| Normality assessment and bootstrap confidence intervals.** Shapiro–Wilk p values (minimum across weather pairs) for the paired differences, and bootstrap 95% confidence intervals (10,000 resamples) of the paired differences vs. the clear condition.

| **Indicator** | **Shapiro_p_min** | **Rain_Δ** | **Rain_CI** | **Snow_Δ** | **Snow_CI** | **Fog_Δ** | **Fog_CI** |
| --- | --- | --- | --- | --- | --- | --- | --- |
| Speed Mean | 0.484 | -0.714 | [-1.536, 0.132] | -0.792 | [-1.789, 0.214] | -1.709 | [-2.435, -0.972] |
| Speed SD | 0.096 | -0.014 | [-0.099, 0.078] | 0.054 | [-0.030, 0.137] | -0.1 | [-0.155, -0.047] |
| RMS Acceleration | 0.042 | 0.014 | [-0.031, 0.062] | 0.041 | [-0.000, 0.084] | -0.04 | [-0.063, -0.017] |
| Steering SD | 0.049 | -0.001 | [-0.002, -0.000] | -0.001 | [-0.002, 0.001] | -0.002 | [-0.004, -0.001] |
| HR Mean | 0.039 | 12.323 | [3.118, 21.838] | 7.362 | [-0.007, 15.400] | 3.139 | [-3.802, 10.324] |
| C4 Spectral Ent. | 0.574 | 0.044 | [0.012, 0.078] | 0.046 | [0.016, 0.075] | 0.038 | [0.009, 0.068] |
| Lateral Offset | 0.002 | 11.505 | [4.129, 19.736] | 9.876 | [4.201, 15.630] | 11.679 | [4.652, 18.949] |
| CORF Pred. Ent. | 0.178 | 0.074 | [0.019, 0.127] | 0.125 | [0.088, 0.161] | 0.099 | [0.048, 0.151] |

**Table S2| CORF model performance.** Per-class precision, recall, F1, accuracy, and expected calibration error (ECE) on out-of-fold predictions (6,456 windows in total).

| **Weather** | **Precision** | **Recall** | **F1** | **N** | **Accuracy** | **ECE** |
| --- | --- | --- | --- | --- | --- | --- |
| Benign | 0.945 | 0.938 | 0.942 | 1614 | 0.938 | 0.153 |
| Rain | 0.923 | 0.918 | 0.92 | 1614 | 0.918 | 0.16 |
| Snow | 0.9 | 0.897 | 0.898 | 1614 | 0.897 | 0.167 |
| Fog | 0.909 | 0.923 | 0.916 | 1614 | 0.923 | 0.17 |

**Table S3| All-channel spectral-entropy weather effects (all eight EEG channels).** r = rank-biserial correlation for the contrast vs. clear; p_FDR = Benjamini–Hochberg-corrected p values; W = Kendall’s coefficient of concordance for the Friedman test.

| **Channel** | **Kendall W** | **p**  **(Friedman)** | **p_FDR**  **(Friedman)** | **r**  **(Rain)** | **p_FDR**  **(Rain)** | **r**  **(Snow)** | **p_FDR**  **(Snow)** | **r**  **(Fog)** | **p_FDR**  **(Fog)** | **mean r**  **(adverse)** |
| --- | --- | --- | --- | --- | --- | --- | --- | --- | --- | --- |
| F4 | 0.056 | 0.166 | 0.166 | 0.175 | 0.349 | 0.385 | 0.046 | 0.115 | 0.542 | 0.225 |
| C3 | 0.069 | 0.102 | 0.117 | 0.291 | 0.187 | 0.359 | 0.057 | 0.261 | 0.253 | 0.303 |
| Fz | 0.161 | 0.002 | 0.009 | 0.272 | 0.187 | 0.535 | 0.007 | 0.205 | 0.353 | 0.337 |
| Cz | 0.077 | 0.074 | 0.099 | 0.385 | 0.092 | 0.464 | 0.016 | 0.374 | 0.126 | 0.407 |
| F3 | 0.118 | 0.014 | 0.038 | 0.175 | 0.349 | 0.501 | 0.01 | 0.19 | 0.353 | 0.288 |
| Oz | 0.078 | 0.072 | 0.099 | 0.272 | 0.187 | 0.295 | 0.109 | 0.314 | 0.176 | 0.293 |
| C4 | 0.08 | 0.067 | 0.099 | 0.411 | 0.092 | 0.531 | 0.007 | 0.362 | 0.126 | 0.435 |
| PZ | 0.165 | 0.002 | 0.009 | 0.419 | 0.092 | 0.58 | 0.007 | 0.392 | 0.126 | 0.464 |

**Table S4| Correct-window control analysis.** Weather contrasts of CORF prediction entropy computed on the 5,933 correctly classified windows only (rank-biserial r with Wilcoxon p).

| **Contrast** | **r** | **p** |
| --- | --- | --- |
| Rain vs Clear | 0.426 | 0.0185 |
| Snow vs Clear | 0.749 | <0.001 |
| Fog vs Clear | 0.558 | 0.0016 |

**Table S5| Driver cluster labels.** Standardized subjective-load (Subj_z) and operational-fluctuation (Fluc_z) scores per driver with the k-means (k = 3) cluster assignment.

| **Subject_ID** | **Subj_z** | **Fluc_z** | **cluster** | **cluster_name** |
| --- | --- | --- | --- | --- |
| 1 | 0.834 | 6.116 | 0 | Strained-reactive |
| 2 | -2.251 | -0.884 | 1 | Stable |
| 3 | 0.269 | 4.299 | 0 | Strained-reactive |
| 4 | -1.478 | -0.757 | 1 | Stable |
| 5 | -0.042 | -2.134 | 1 | Stable |
| 6 | -0.883 | -1.378 | 1 | Stable |
| 7 | 3.037 | -0.408 | 2 | Tense-compensating |
| 8 | 0.113 | -1.768 | 1 | Stable |
| 9 | -3.38 | -0.286 | 1 | Stable |
| 10 | -1.732 | -0.178 | 1 | Stable |
| 11 | 0.819 | 0.815 | 2 | Tense-compensating |
| 12 | 0.077 | -1.202 | 1 | Stable |
| 13 | 1.594 | -0.175 | 2 | Tense-compensating |
| 14 | -0.839 | -0.465 | 1 | Stable |
| 15 | -0.938 | 0.609 | 1 | Stable |
| 16 | 0.058 | -0.729 | 1 | Stable |
| 17 | -0.537 | 1.884 | 0 | Strained-reactive |
| 18 | -0.27 | -2.361 | 1 | Stable |
| 19 | 0.126 | -2.467 | 1 | Stable |
| 20 | -0.141 | 2.09 | 0 | Strained-reactive |
| 21 | 1.189 | -0.773 | 2 | Tense-compensating |
| 22 | -1.063 | -0.97 | 1 | Stable |
| 23 | -1.191 | -2.442 | 1 | Stable |
| 24 | -0.26 | -0.272 | 1 | Stable |
| 25 | 1.611 | -0.781 | 2 | Tense-compensating |
| 26 | 0.734 | 3.314 | 0 | Strained-reactive |
| 27 | 1.316 | 1.582 | 2 | Tense-compensating |
| 28 | 0.599 | 0.565 | 2 | Tense-compensating |
| 29 | 2.042 | -1.294 | 2 | Tense-compensating |
| 30 | 0.586 | 0.45 | 2 | Tense-compensating |

**Data S1|** Aggregated participant × weather dataset (120 rows) supporting all statistical analyses, provided as a separate CSV file (数据S1_被试级汇总数据_120点.csv).

**Code|** The analysis code reproducing all supplementary tables and figures (Supplementary_Analysis_Code.py) is provided as a separate file.
